# Presence 5 for Racial Justice Workshop: Fostering Dialogue Across Medical Education to Disrupt Anti-Black Racism in Clinical Encounters

**DOI:** 10.15766/mep_2374-8265.11227

**Published:** 2022-02-10

**Authors:** Megha Shankar, Kelsey Henderson, Raquel Garcia, Gabrielle Li, KeAndrea Titer, Rhonda Graves Acholonu, Utibe R. Essien, Cati Brown-Johnson, Joy Cox, Jonathan G. Shaw, Marie Christine Haverfield, Kenji Taylor, Sonoo Thadaney Israni, Donna Zulman

**Affiliations:** 1 Advanced Physician Fellow, Center for Innovation to Implementation, Veterans Affairs Palo Alto Health Care System; Postdoctoral Scholar, Center for Health Policy/Center for Primary Care and Outcomes Research, Stanford University; 2 Second-Year Medical Student, Meharry Medical College; 3 Fourth-Year Undergraduate Student, San Jose State University; 4 Fourth-Year Undergraduate Student, Stanford University; 5 Assistant Professor, Department of Medicine and Hospital Medicine, University of Alabama at Birmingham; 6 Associate Professor of Pediatrics, Division of Hospital Medicine, Department of Pediatrics, Children's Hospital at Montefiore/Albert Einstein College of Medicine; 7 Assistant Professor of Medicine, Division of General Internal Medicine, Department of Medicine, University of Pittsburgh; Core Investigator, Center for Health Equity Research and Promotion, Veterans Affairs Pittsburgh Healthcare System; 8 Research Scientist, Division of Primary Care and Population Health, Stanford University; 9 Program Development Analyst, Office of Primary Care and Community Initiatives, Rutgers New Jersey Medical School; Presence Fellow, Presence Center, Stanford University; 10 Clinical Associate Professor, Division of Primary Care and Population Health, Stanford University; 11 Assistant Professor, Department of Communication Studies, San Jose State University; 12 Stanford Intermountain Fellow and Instructor, Division of Primary Care and Population Health, Stanford University; 13 Executive Director, Presence Center, Stanford University; 14 Associate Professor, Division of Primary Care and Population Health, Stanford University; Associate Director, Center for Innovation to Implementation, Veterans Affairs Palo Alto Health Care System

**Keywords:** Physician-Patient Relations, Medical Humanities, Diversity, Inclusion, Health Equity, Anti-racism

## Abstract

**Introduction:**

Anti-Black racism has strong roots in American health care and medical education. While curricula on social determinants of health are increasingly common in medical training, curricula directly addressing anti-Black racism are limited. Existing frameworks like the Presence 5 framework for humanism in medicine can be adapted to develop a novel workshop that promotes anti-racism communication.

**Methods:**

We performed a literature review of anti-racism collections and categorized anti-racism communication practices using the Presence 5 framework to develop the Presence 5 for Racial Justice Workshop. Implementation included an introductory didactic, a small-group discussion, and a large-group debrief. Participants evaluated the workshop via an online survey, and we analyzed the resulting qualitative feedback.

**Results:**

A total of 17 participants took part in two workshops, with nine of the participants responding to the evaluation survey. Themes that emerged from survey responses included strengths of and improvements for the workshop structure (protected time for anti-racism discussion, dialogue between learners and faculty) and content (specific phrases and language, practicing self-reflection).

**Discussion:**

The workshop provides participants with a semistructured discussion around the five anti-racism communication practices. Barriers to implementation include incorporating the workshop into existing curricula and ensuring diverse learners. Barriers to evaluating the workshop include the low survey response rate. Recommendations to improve the workshop include using case-based discussion and varying the workshop structure according to institutional needs. Next steps include an implementation study to evaluate the acceptability, feasibility, and effectiveness of the workshop.

## Educational Objectives

By the end of this activity, learners will be able to:

1. Learn the five evidence-based Presence 5 for Racial Justice practices for anti-racism in clinical education.
2. Discuss how to apply Presence 5 for Racial Justice practices for anti-racism communication in clinical training.
3. Provide examples and specific phrases/language for each of the Presence 5 for Racial Justice practices.

## Introduction

Anti-Black racism has strong roots in medicine and medical education.^1^ Open discussions of racism across training levels—beyond awareness of implicit bias and health disparities—are critical to educating a generation of physicians committed to anti-racism.^2^ While curricula on social determinants of health and diversity, equity, and inclusion (DEI) are increasingly common in medical education,^3^ curricula that directly address racism are limited, and experts have called for more specific, intentional, required anti-racist curricula that demonstrate an investment in and commitment to racial justice.^4^ Furthermore, there is a gap in the literature describing theory-driven and evidence-grounded interventions that address anti-Black racism specifically and in a way that fosters dialogue between trainees, creating an opportunity for rigorously developed interventions that facilitate focused, nuanced discussions during medical education.

Race is socially constructed, and racism is a belief in racial hierarchy.^5^ Directed against Black individuals, anti-Black racism devalues, dehumanizes, and systematically marginalizes Black individuals.^6^ Anti-Black racism is unique given the context of trans-Atlantic slavery in the United States, which set a foundation of structural racism leading to anti-Black policies, requiring a specific focus towards reconciliation. Events in 2020, including George Floyd's murder and the co-occurrence of marked racial health inequities, prompted a reckoning on anti-Black racism and the need for anti-racism communication that focuses on the unique lived experiences of Black patients.^7^ Urgent changes are needed to work towards racial health equity for Black patients, starting by teaching learners how to combat anti-Black racism directed at Black patients by the health care system and clinicians.

Dismantling racism in medicine requires that medical education promote racial justice throughout clinical training, longitudinally integrating interventions into existing curricular structures to foster deep reflection and difficult dialogue among learners of all levels. One approach to filling the gap of anti-racism training in medical education is through an intervention that is rooted in interpersonal connection and humanism, a value that spans the ACGME six core competencies.^8^ Humanism-focused frameworks like the Presence 5^9^ may be an effective approach in teaching anti-racism communication because they center the patient within the patient-physician relationship. Originally designed for clinicians and intended to address technology and administrative demands of clinical practice as well as to reduce medical errors, the Presence 5 framework seeks to foster meaningful connections between the patient and clinician by focusing on the human aspect of medicine. The Presence 5 framework fosters clinician humanism through tangible, evidence-based, interpersonal practices across five domains: (1) prepare with intention, (2) listen intently and completely, (3) agree on what matters most, (4) connect with the patient's story, and (5) explore emotional cues.^9^ These practices are disseminated and implemented in clinical settings through discussion groups where clinicians learn about and share how they practically use the Presence 5 to promote humanism in clinical care.

Given a strong evidence base and practical application, Presence 5 is an ideal framework to serve as a foundation for urgently needed anti-racism training in clinical practice. Therefore, we sought to adapt the Presence 5 framework to anti-racism in medical education through a literature review and an external review by national experts in DEI to develop the Presence 5 for Racial Justice Workshop. This workshop offers a novel approach to teaching anti-racism with the following three critical features: The workshop is (1) discussion based, promoting open dialogue among diverse learners; (2) evidence based, ensuring recommended practices are rooted in the literature; and (3) practical, providing learners with usable anti-racism strategies in everyday clinical care. The Figure shows the Presence 5 for Racial Justice practices, which can be summarized as follows:

1. Prepare with intention: reflect on identity and power dynamics,^10^ prepare by learning established frameworks to address racism in the moment.^11^
2. Listen intently and completely: listen for racism-related factors influencing health without interrupting.^12^
3. Agree on what matters most: allow patients to direct the conversation around racism and health.^13–15^
4. Connect with the patient's story: learn about a patient's experiences with racism to promote dialogue on care plans,^16^ identify barriers beyond a disease,^17^ and encourage patient advocacy^11^ to eliminate stigmatizing language around a patient's race.^18^
5. Explore emotional cues: reflect, validate, and confirm a patient's emotions around racial trauma.^19^

In this preliminary implementation of the Presence 5 for Racial Justice Workshop, we describe lessons learned from an evaluation of two workshops.

**Figure.**
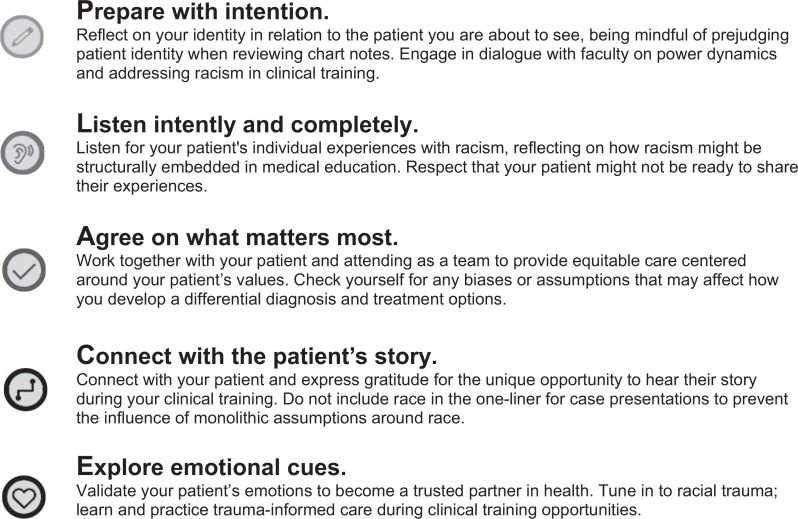
Presence 5 for Racial Justice Workshop: practice overview.

## Methods

The Presence 5 for Racial Justice Workshop was developed through a literature review and external review by national experts in medical education and health equity. All authors agreed on the final wording and format of the workshop. This discussion-based workshop was intended for learners of diverse racial backgrounds and levels of medical training to foster meaningful conversation. This project was approved by the Stanford Institutional Review Board (protocol #57663) on August 17, 2020.

### Implementation

We implemented the Presence 5 for Racial Justice Workshop through a virtual, 1-hour workshop via Zoom. The workshop was facilitated by a faculty member, fellow, and medical student to encourage dialogue across training levels. Facilitators reviewed the Presence 5 for Racial Justice Workshop guide (Appendix A) and the introductory didactic slides (Appendix B) thoroughly prior to facilitation. There was also an optional 30-minute train-the-trainer video module with guidance around facilitation. There were no prerequisites for participation in the workshop.

#### Introductory didactics

We began the workshop with a 15-minute didactic portion, during which we used screenshare functionality to show slides (Appendix B) reviewing the learning objectives, an overview of the Presence 5 framework, and the importance of adaptation for racial justice. We then presented detailed evidence followed by the associated Presence 5 for Racial Justice practices. At this time, participants were provided with the Presence 5 for Racial Justice Workshop guide (Appendix A) and postdiscussion mental health resources (Appendix C) via a link in the virtual chat. We emphasized to participants that discussions around racism could be difficult, which was why we were providing the mental health resources (i.e., websites, hotlines, groups) to help them navigate these conversations in a culturally appropriate manner. We also reminded participants to reach out to facilitators for any immediate concerns.

#### Small-group discussion

We used three breakout rooms for a 35-minute discussion, assigning facilitators to each breakout room and evenly distributing participants. We encouraged participants to turn their video on during the breakout groups with a stated goal of increasing engagement and dialogue. In the breakout rooms, we began by reading aloud the ground rules for discussion, beginning with “Racism is a challenging subject matter,” to encourage participants to respect each other and also to remind them of the mental health resources available in Appendix C. We then conducted a self-reflection exercise, asking participants to write a positionality statement^12^ reflecting on various aspects of their identity and what perspectives they might bring to a patient encounter.

Next, we presented a case, stopped the screenshare, and spent the remainder of the small group facilitating discussion on each of the five practices using the discussion questions in the Presence 5 for Racial Justice Workshop guide (Appendix A). Participants shared how they might apply the Presence 5 for Racial Justice practices as well as offering personal anecdotes of practicing anti-racism in medicine. Participants discussed important and nuanced concepts such as anti-Black racism, identity, power dynamics, bias, and microaggressions in the context of clinical training and patient care. At the end of the discussion, participants set one or more concrete goals to incorporate a specific anti-racism communication practice in their next clinical encounter. We screenshared the goal setting and action plan structure.

#### Large-group debrief

For this portion of the workshop, participants were brought back into the main virtual room to share their goals and reflections from each of the small groups. Participants shared reflections aloud, as well as in the virtual chat. At the end of the workshop, we reminded participants of the mental health resources previously provided (Appendix C) given the sensitive nature of this topic and then concluded by providing the evaluation survey link in the chat (Appendix D).

### Evaluation

Completion of the evaluation survey was voluntary. We analyzed demographic data from participants using summary statistics, and we iteratively and systematically integrated qualitative survey feedback using a thematic content approach,^20^ adding suggested practices, references, and teaching strategies.

## Results

A total of 17 participants completed the Presence 5 for Racial Justice Workshop. Seven internal medicine residents completed the workshop during an institutional didactic session, and a combination of 10 medical students, residents, fellows, and faculty completed the workshop during a regional general internal medicine conference. Nine of 17 workshop participants responded to the evaluation survey, for a response rate of 53%. The respondents were of diverse backgrounds, including gender, race, ethnicity, level of training, clinical setting, and DEI experience (Table).

**Table.**
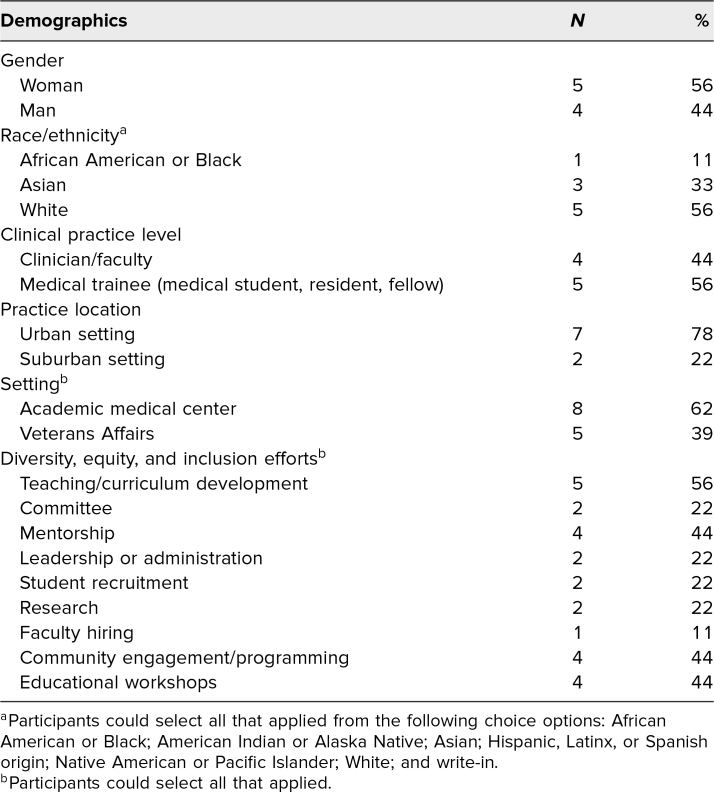
Presence 5 for Racial Justice Curriculum Workshop Participant Demographics (*N* = 9)

### Workshop Results

The survey focused on level 1 (reaction) of Kirkpatrick's four levels of evaluation,^21^ with questions soliciting feedback on each of the Presence 5 for Racial Justice practices as well as workshop content and structure. Emergent themes included strengths of and improvements for workshop content and structure.

Participants recommended improvements to the Presence 5 for Racial Justice practices by providing additional suggestions of anti-racism communication practices, specific language/phrases to use in clinical training, and additional literature, all of which were incorporated into the final workshop.

#### Workshop content

Participants identified strengths of and improvements for the Presence 5 for Racial Justice Workshop content. Participants identified the guidance regarding how to have difficult conversations with patients about racism and health as a strength of the discussion. One participant commented that a helpful part of the workshop was “listening to the personal experiences we have with interacting with Black patients and stating our biases when it came to these experiences.” Another participant noted, “Advice on how to have these important conversations with patients was the most helpful part of the discussion, and the time and space to reflect on disparities and solutions to inequities was helpful.” Participants noted that the case provoked good discussion, and they appreciated sharing and listening to personal experiences about practicing anti-racism in clinical encounters.

To improve the workshop content, participants noted that it might be helpful to provide prompts of possible patient comments around the topic of racism during clinical encounters coupled with discussion around how to respond to these comments.

#### Workshop structure

Regarding workshop structure, participants appreciated the organization of the workshop, highlighting the utility of the introductory didactic portion as well as the case-based, small-group discussion and goal setting. Participants appreciated the evidence-driven didactic portion of the workshop; one participant commented, “I appreciated the inclusion of data in the introduction materials.” Regarding improvement for the workshop structure, participants recommended having more protected time and more opportunities for discussions around racism and medicine.

## Discussion

We successfully implemented the Presence 5 for Racial Justice Workshop to provide participants across training levels with the time and space for a semistructured, evidence-based, and insightful discussion around practicing anti-racism in medicine. We believe this is the first adaptation of a humanism-based framework to develop a workshop on anti-racism communication for diverse learners. The overall objectives were met, and this novel workshop adds to the growing and evolving repository of medical education efforts dedicated to addressing racism in medicine.

### Limitations

There were barriers to implementing the Presence 5 for Racial Justice Workshop. The absolute number of participants was low due to lower virtual conference attendance rates. Similarly, the number of participants who completed the survey was low (*N* = 9), likely due to increasing demands during the COVID-19 pandemic. As this was a preliminary project to enable a larger implementation study, we solely focused on level 1 of Kirkpatrick's four levels of evaluation.^21^ Due to pandemic restrictions, these workshops were delivered virtually, which may have limited some aspects of learner engagement. The benefit of the virtual nature of these workshops, however, was the opportunity to involve a geographically diverse group of participants, which added to the richness of the discussion.

Participants noted the importance of carving out time for regular and ongoing discussions around anti-racism, yet a major barrier was finding the time within existing curricula during medical training. Several institutions have followed national recommendations around developing anti-racism medical education curricula,^22^ but it is unclear if these will be lasting and sustainable efforts.

Finally, the Presence 5 for Racial Justice Workshop provided communication practices to address anti-Black racism aimed towards patients by the health care system and clinicians. While this is a crucial step towards health equity, there is a need for trainings aimed at addressing anti-Black racism towards Black learners in medical education, whether coming from patients, other learners, or faculty. This is vital to creating a culture of inclusion as well as supporting the pathway of Black learners into health care fields.^23^

### Recommendations

We recommend implementing this workshop using a case-based discussion. Participants appreciated the case as a practical way to spark discussion around anti-racism practices in the clinical setting. We also recommend all workshop facilitators review Appendices A and B and undergo the optional Presence 5 for Racial Justice Workshop train-the-trainer module (video link in Appendix B)—this preparation can take about an hour in total. Facilitators can be chosen based on available resources and personnel, and there is an opportunity for peer-to-peer facilitation with learners (such as upper-level medical students or senior residents) as workshop facilitators. In choosing facilitators, it is important to be mindful of the minority tax^24^ so as not to overburden facilitators of color with the task of teaching anti-racism communication in medicine. We recommend emphasizing the evidence base as the foundation of this workshop, as participants appreciated this as a major strength of the workshop.

There may be various ways to structure this workshop, depending on institutional resources; given the time and depth needed for discussion on these important topics, the workshop could include a five-part series with discussions for each of Presence 5 for Racial Justice practices. Alternatively, small groups could talk about one Presence 5 for Racial Justice practice each and share reflections through a teach-back with the larger group.

### Future Directions

To further evaluate the Presence 5 for Racial Justice Workshop, an implementation study is under development within undergraduate and graduate medical training. Evaluations will consider the acceptability and feasibility of the discussion circles format, as well as the effectiveness of building knowledge, skills, and confidence around anti-racism communication practices. The long-term goals of implementing Presence 5 for Racial Justice Workshop within medical education are to transform the culture and practice of medicine, foster humanism in clinical encounters, and promote health equity for Black patients.

## Appendices


Presence 5 for Racial Justice Guide.docxIntroductory Didactic.pptxParticipant Resources.docxSurvey.docx
*All appendices are peer reviewed as integral parts of the Original Publication.*
