# Supplementary material for: Presence 5 for Racial Justice Workshop: Fostering Dialogue Across Medical Education to Disrupt Anti-Black Racism in Clinical Encounters
Source: MedEdPORTAL. 2022 Feb 10;18:11227. doi: 10.15766/mep_2374-8265.11227 (PMC8828658; doi:10.15766/mep_2374-8265.11227)
Supplement: Supplementary file 1 — Presence 5 for Racial Justice Guide.docxIntroductory Didactic.pptxParticipant Resources.docxSurvey.docx [file mep_2374-8265.11227-s001.zip › C. Participant Resources.docx]

# Presence 5 for Racial Justice Participant Resources

Discussing racism is challenging, and everyone has different ways to care for themselves. This list provides various approaches to seeking professional care, individual self-care exercises, and more. Feel free to use what you feel is most beneficial to you. (Web-links provided are optional).

**Resources Centering Black Individuals and Other Individuals of Color**

# *National Services/Hotlines*

- [BlackLine](https://www.callblackline.com/): A 24/7 national crisis support hotline (with a text option as well) that provides a space for peer support and counseling, reporting of mistreatment, and affirming the lived experiences to those who are most impacted by systematic oppression. Operated with an LGBTQ+ Black Femme affirming lens. 1(800) 604-5841

*Black Men*

- [Black Men Heal](https://blackmenheal.org/): Provides free access to mental health treatment, psycho-education, individual psychotherapy or group therapy, training and consultations, and community

resources to men of color. They also offer “King's Corner,” which is a virtual safe meetup

for men held every Sunday evening via Zoom

- [Therapy for Black Men](https://therapyforblackmen.org/): TherapyForBlackMen.org is a directory to help men of color find a therapist. Using the directory, men can search by therapist location, specialization, and many other criteria. Searching by location, the results will include the therapists near you and will display their credentials, location, and the issues they treat. They will soon be supporting free therapy sessions for Black Men who do not have insurance or Medicaid/Medicare

*Black Women*

- [Dear Black Women](https://www.dearblackwomenproject.com/): An affirmation movement for black women by black women that provides therapy resources, daily affirmations, tips, mind & body resources, affirming reads and podcasts
- [Therapy for Black Girls](https://providers.therapyforblackgirls.com/): Website with a directory to find trusted, culturally competent therapists for Black women, as well as a blog, podcast, and community

*QTPOC*

- [Going to therapy as a QTPOC, without being harmed, erased or baffled](https://www.bgdblog.org/2014/12/going-therapy-qtpoc-without-harmed-erased-baffled-therapy-horror-stories-may-heard/): Article providing information and tips for seeking therapy as a QTPOC
- [National Queer and Trans Therapists of Color Network](https://www.nqttcn.com/directory): Interactive digital resource that helps QTPoC locate QTPoC mental health practitioners across the United States

*Meditation*

- [L](https://liberatemeditation.com/)iberate Meditation App: Free IOS/Android meditation app for Black individuals, made by Black individuals
- [Black Lives Matter Meditation for Healing Racial Trauma](https://www.drcandicenicole.com/post/black-lives-matter-meditations): A 17-minute guided meditation provided by Dr. Candice Nicole using mindfulness, affirmation, and metta for racial trauma.

*Recorded Webinar with Strategies for Coping/Self-Care*

- [The Effects of Racism on Mental Health: How to Cope](https://adaa.org/webinar/consumer/effects-racism-mental-health-how-cope): Recorded webinar focused on the negative impact of racism on mental health symptoms for people of color; provides some coping resources to deal with the stress, anxiety, and overall emotional toll of racism

*Community Resources*

- [Ourselves Black](https://ourselvesblack.com/online-magazine): Website containing mental health resources, a provider directory, mental illness library, podcast, and blog. Ourselves Black is also a biannual mental health resource magazine for the Black community, written and designed to focus on exploration of our mental selves through a variety of engaging resources, interviews, stories, and photography to promote Black mental health.
- [Black Emotional and Mental Health (BEAM)](https://wellness.beam.community/): Provides a variety of toolkit and resources for coping, supporting, self-control, and emotional awareness; directory for telehealth therapy
- [Inclusive Therapists](https://www.inclusivetherapists.com/reduced-fee-virtual-teletherapy): Reduced fee tele-therapy led by Black and Indigenous therapists
- [Black Mental Health Alliance](https://blackmentalhealth.com/connect-with-a-therapist/): Virtual directory of culturally-competent and patient- centered licensed mental health professionals
- [Boris Lawrence Henson Foundation Resource Directory](https://borislhensonfoundation.org/resource-guide/wpbdp_category/therapist/): Virtual directory of mental health providers, programs and resource materials for the African American community

*Blog/Social Media*

- [Black Girl + Mental Health Blog](https://blackgirlmentalhealth.tumblr.com/): Blog on mental health resources and tips specifically for Black women and girls.
- [Cultured Therapy](https://www.instagram.com/culturedtherapy_/)- Instagram account that provides mental health resources and tips that center Black communities
- [A Grief and Honor Playlist](https://open.spotify.com/playlist/2svDyF1kaXSBV4LKMdkVFk?si=k7S8Mg02QqmsZpo_5m65sw) of Black artists curated by a group of University of Michigan Black Alumni and started Christian Ziraldo

**Additional Resources**

# *National Services/Hotlines*

- Text 741741 from anywhere in the U.S. to chat with a trained crisis counselor through [Crisis Text Online](https://www.crisistextline.org/text-us/)
- [Crisis Link](https://prsinc.org/crisislink-hotline-textline/) provides 24/7 crisis intervention, suicide prevention and support to foster self-sufficiency by providing problem-solving skills and information on community resources so callers can better cope with emotional trauma, personal and family crises. Text "CONNECT" to 855-11 or call 703.527.4077.

*Community*

- [Ethel’s Club](https://www.ethelsclub.com/): Digital community designed for people of color to tune into daily classes across wellness & culture, make connections with a thriving community, and access free mental health counseling resources in an online community designed for people of color to thrive. Hosts free, hour-long grieving sessions twice monthly. Led by licensed Black therapists to help hold space and process the weight of the many complex emotions that we are feeling and carrying right now.
- [Talk Space](https://www.talkspace.com/): Online and mobile therapy company. Users have access to licensed therapists through the website or mobile app. Therapeutic services can be provided via text, video, and/or audio messaging. Includes “Talkspace Support Groups” that are free, therapist-led support groups for coping with racial trauma

*Articles & Toolkits*

- [100 Radical Acts of Self-Care](https://mcusercontent.com/6aa11fa786466642f2188db2e/files/b0f976a5-31fd-4406-9033-b31620907758/100_Self_Care_Ideas_by_GirlTrek_compressed.pdf): This guide provides a list of things that allow Black women to take time to rest, reflect, and heal to consider their own needs or prioritize time for self-care.
- [Talking about Race: Self-Care](https://nmaahc.si.edu/learn/talking-about-race/topics/self-care): Article describing and providing concrete practices for self-care, meditation, mindfulness, reflection, and positive affirmations
- [Family Care, Community Care, and Self-Care Toolkit](http://www.abpsi.org/pdf/FamilyCommunitySelfCareToolKit.pdf): Healing in the Face of Cultural Trauma: Pdf toolkit with strategies for self-care, community-care, family-care, and coping with cultural trauma. This toolkit also provides a facilitator’s guide for hosting healing circles. (English/Spanish)
- [Grief is a direct impact of racism: Eight ways to support yourself](https://theconversation.com/grief-is-a-direct-impact-of-racism-eight-ways-to-support-yourself-91750): Article with eight strategies for self-care through grief
- [#RacialTraumaIsReal](https://www.bc.edu/content/dam/files/schools/lsoe_sites/isprc/pdf/racialtraumaisrealManuscript.pdf): Manuscript describing and defining racial trauma, detailing racism recovery plan steps, and providing strategies from mental health professionals and advocates of the study and promotion of racial and cultural understanding and discussion

*Meditation*

- [Do Nothing Tool](http://www.donothingfor2minutes.com/): Provides a 2-minute timer for relaxation and meditation.

# Local/Clinic Resources: Each institution and clinic may have local resources – please ask your facilitator about these (e.g., social work, community health worker, support groups, on-site mental health professional, etc.).
