# Supplementary material for: Presence 5 for Racial Justice Workshop: Fostering Dialogue Across Medical Education to Disrupt Anti-Black Racism in Clinical Encounters
Source: MedEdPORTAL. 2022 Feb 10;18:11227. doi: 10.15766/mep_2374-8265.11227 (PMC8828658; doi:10.15766/mep_2374-8265.11227)
Supplement: Supplementary file 1 — Presence 5 for Racial Justice Guide.docxIntroductory Didactic.pptxParticipant Resources.docxSurvey.docx [file mep_2374-8265.11227-s001.zip › A. Presence 5 for Racial Justice Guide.docx]

Presence 5 for Racial Justice Workshop

*An anti-racism discussion session to promote health equity*


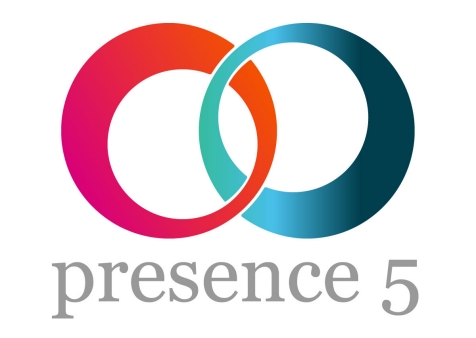


(Image is author owned)

**Learning Objectives**

By the end of this activity, learners will be able to:

1. Learn the 5 evidence-based *Presence 5 for Racial Justice* practices for anti-racism in clinical education.

2. Discuss how to apply *Presence 5 for Racial Justice* practices for anti-racism communication in clinical training.

3. Provide examples and specific phrases/language for each of the *Presence 5 for Racial Justice* practices.

**Overview**

Medical education requires a transformation to promote racial justice in clinical care and interactions. Anti-Black racism in medicine has a long history and seeps into clinical care and interactions between patient, attending, medical trainee, and associated healthcare workers. The majority of medical trainees believe that current medical education fails to equip them with the tools to adequately address racism in medicine. The purpose of the *Presence 5 for Racial Justice Workshop* is to provide medical students, residents, and fellows a space to share and discuss anti-racism communication practices. This discussion-based workshop seeks to help trainees un-learn, reflect, and re-learn how to promote racial justice through medicine.

When a trainee and a patient make a genuine connection during clinical encounters, both benefit. Combating anti-Black racism in medicine can foster meaningful and trusting connection in today’s demanding clinical world, while improving health outcomes for Black patients. Anti-racism strategies can allow trainees to better advocate for Black patients, be more present and engaged, and feel more grounded and energized at work despite the challenges of clinical practice. The *Presence 5 for Racial Justice Workshop* is an evidence-based framework for practices that have been shown to generate more trusting clinical encounters for both clinicians and patients.

This is not an all-encompassing guide for anti-racism in medicine; we hope that this will spark discussion, awareness, and accountability in medical education. These practices are not a fix-all solution to racism in medicine; rather, they are evidence-based suggestions that can be employed in the life-long commitment to be present for racial justice.

**Ground Rules**

- **Discussion is confidential and judgment-free.** Focus on discussing ideas, rather than people.
- **Respect others** and embrace discomfort and disagreement. Racism is a challenging subject-matter.
- **Be thoughtful in when you choose to speak vs listen.** Be considerate of what you are asking of others. There is no pressure to share.
- **We all have biases**. If you experience or witness racism in today’s conversation, voice your concerns as you feel comfortable and seek to recognize your blind spots. Remember that everyone is evolving.
- **Assume positive intent.** Racism is sometimes unintentional. Be open to dialogue about the nuances without making it personal.
- **Acknowledge each other’s unique identity and circumstances**. Avoid stereotyping or talking about groups as monolithic and be mindful of the “minority tax” – avoid placing an undue burden of work on minority individuals. Rely on the evidence in this guide.
- **Focus on solutions rather than problems,** when possible.
- **Put away distractions.** Be fully present for the conversation.
- *What other ground rules would you like to include today?*

**Self-Reflection**

Write a positionality statement. This is a few sentences about your identity – privilege, oppression, position in society – and the perspective you have in this discussion and the perspective you bring to clinical encounters.

**Case for Discussion**

You are about to see a new patient. Her chart indicates that she is a 35-year-old Black woman who has hypertension. She has many high blood pressure readings in the chart. Notes suggest that she is "non-compliant" with her medications and has a "complex social history.“ Physicians have tried several different medications on her without improvement of her blood pressure readings. Some notes mention she does not trust the medical system and feels she is being experimented on with so many medications.

**Presence 5 for Racial Justice Practices**

|  |  | **Prepare with intention** | **Examples** |
| --- | --- | --- | --- |
|  |  | Reflect on your identity in relation to the patient you are about to see, being mindful of pre-judging patient identity when reviewing chart notes. Engage in dialogue with faculty on power dynamics and addressing racism in clinical training.  **Are you prepared for a meaningful and racially just interaction?** | - Consider how your identity influences biases in patient interactions - Become familiar with respectful strategies to address bias and racism in the moment and discuss how to navigate power dynamics with mentors when addressing racism - Review your patient’s social history and recognize racism as a determinant of health |
|  |  | **Listen intently and completely** | **Examples** |
|  |  | Listen for your patient's individual experiences with racism, reflecting on how racism might be structurally embedded in medical education. Respect that your patient might not be ready to share their experiences.  **When discussing racism and health, what does your patient say when uninterrupted?** | - Listen for your patient’s experiences with racism, bias, or mistreatment - As you listen, remember how systemic racism in medical education (e.g., underrepresentation of Black skin in clinical images) influences clinical decisions - Seek patient permission when inquiring about instances of racism and trauma in healthcare |
|  |  | **Agree on what matters most** | **Examples** |
|  |  | Work together with your patient and attending as a team to provide equitable care, centered around your patient’s values. Check yourself for any biases or assumptions that may affect how you develop a differential diagnosis and treatment options.  **How can you and your patient be on the same team without the interference of assumptions or biases?** | - Reflect on bias or assumptions that come up and create a plan towards health equity by including the patient fully in decision-making - Allow the patient to describe in their own words their plan and answer any questions, advocating for their best care possible - Celebrate effort and progress using positive language to promote trust and partnership |
|  |  | **Connect with the patient’s story** | **Examples** |
|  |  | Connect with your patient and express gratitude for the unique opportunity to hear their story during your clinical training. Do not include race in the “one-liner” for case presentations to prevent the influence of monolithic assumptions around race.  **How do you combat anti-Black racism in your patient’s health journey?** | - Invite your patient to share their story to the level of their comfort and consider historical instances of racism that may play a role - Avoid including information about race when presenting, unless it is relevant to social determinants of health - Consider appropriate referrals to interdisciplinary staff when indicated by the patient’s presentation |
|  |  | **Explore emotional cues** | **Examples** |
|  |  | Validate your patient’s emotions to become a trusted partner in health. Tune in to racial trauma; learn and practice trauma-informed care during clinical training opportunities.  **What can you learn from your patient’s emotions to address racial trauma?** | - Recognize racial trauma and ask to confirm what emotion you see or hear and follow up on this - Be careful to not label your patients’ emotions based on racial stereotypes - Explicitly state how your patient’s emotions and experiences impact their health |

(Icons are author owned)


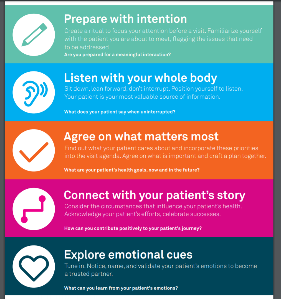

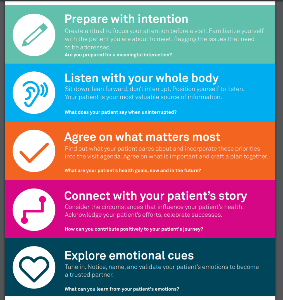

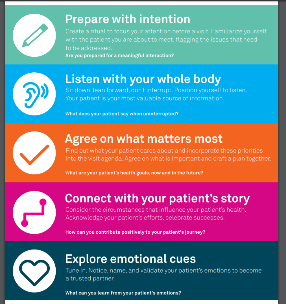

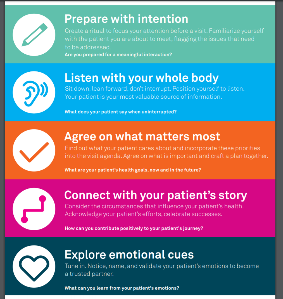

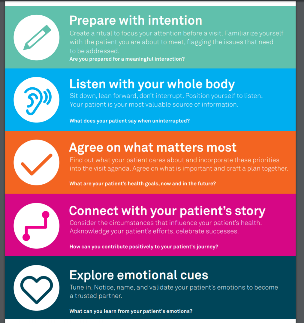
 Evidence Review & Discussion

# **
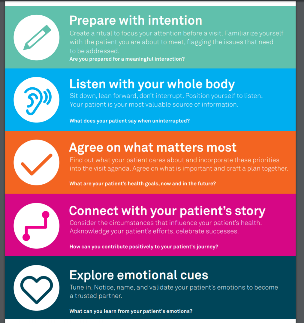
Prepare with intention**

**Discussion Questions**

- What do you do to ‘Prepare with Intention’ for your clinical interactions?
- What practices do you keep on hand to address racism in the moment?
- How have you navigated power dynamics within the structure of medical education that may influence your ability to provide racially just care? Have there been times where an attending behaves in a way that is racially unjust towards a patient?

**Discussion Questions**

- What do you do to ‘Prepare with Intention’ for your clinical interactions?
- What practices do you keep on hand to address racism in the moment?
- How have you navigated power dynamics within the structure of medical education that may influence your ability to provide racially just care? Have there been times where an attending behaves in a way that is racially unjust towards a patient?

# **Evidence suggests:**

# When medical students reflect on the diversity of their social communities and identify what elements are key to their personal identity, they were more likely to demonstrate understanding of how their social identities can impact clinical decision-making and social inequities (Chow 2019).

# Race is a social construct that has been incorrectly used as a proxy for pathology in medical education, propagating institutionalized racism (Nieblas-Bedolla 2020)

# Following a sustained practice of self-reflection regarding implicit biases and engaging in conversations about racism amongst colleagues can begin to reduce bias and racism in clinical settings (Bailey 2017, Tsai 2018)

# Open conversations between faculty and trainees on power dynamics can empower trainees in the face of microaggressions (Acholonu 2020). During clinical orientations, discuss how to address racism and racial trauma in clinical interactions before they happen (Shankar 2019)

# Engaging in discussions of hypothetical scenarios related to bias and identifying non-threatening strategies to address bias leads to increased confidence in addressing instances of bias when witnessed and recognized (DallaPiazza 2018)

# Consider using the CHARGE2 framework to mitigate your own bias (DallaPiazza 2018)

| C—Change your context: Is there another perspective that is possible? |
| --- |
| H—Be Honest: With yourself, acknowledge and be aware. |
| A—Avoid blaming yourself: Know that you can do something about it. |
| R—Realize when you need to slow down. |
| G—Get to know people you perceive as different from you. |
| E—Engage: Remember why you are doing this. |
| E—Empower patients and peers. |

# **Evidence-based practices:**

- **Take stock** **of your own privileges**, oppressions, and biases in relation to the patient you are about to see. What values and perspectives are you bringing to the visit? **Think about how your patient may perceive you**. (e.g. Have similarly-looking clinicians been triggering personally or historically for the patient? What harms has your white coat done historically?)
- **Continue to challenge yourself to learn and reflect** on your understanding of race and racism. This an ongoing learning process of introspection and self-interrogation. Consider journaling, peer dialogues, reading and reflecting on literature around racism in medicine.
- **Reflect on how diverse** your personal network and educational community are compared to the patients you treat; do your patients look like you and those you typically interact with? Make an effort to expand your network to interact with people who are different than you.
- **Acknowledge power dynamics** in the clinical interactions between you, attending, and patient. If you observe an attending discriminating against patient, consider asking the attending, **“I’m curious, what makes this case different from last week? How do you determine these changes in treatment plans?”**
- **Be mindful of pre-judging patient identity while performing chart review.** Pause to reflect when reading descriptors such as “non-compliant” or descriptors about race.

# **
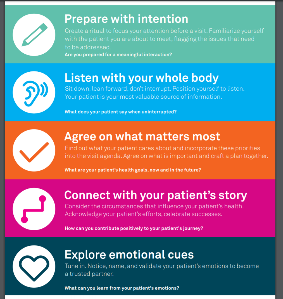
 Listen intently and completely**

**Discussion Questions**

- What do you do to ‘Listen Intently and Completely’ to you patients in your clinical interactions?
- How do you listen to your patients with an open mind while acknowledging gaps in clinical training (e.g., underrepresentation of Black skin in clinical images)?
- How do you ensure active listening, especially when patients discuss racial trauma?

# **Evidence suggests:**

# When physicians meet with Black patients, they are significantly more likely to have shorter visits, speak faster, and verbally dominate the conversation (Cooper 2012, Johnson 2004).

# Asking patient-centered questions, such as “How does your medical condition affect your life or your family’s lives?” allows patients the space to share the social context surrounding their health concerns and facilitates a trusting partnership with the patient (Batista 2018, Drake 2017)

# Recognizing and addressing one’s own bias while listening to the patient’s concerns and story can improve equity and quality of patient care (Medlock 2017).

# Listen to your patient as the primary way to inform your assessment; considering broad differential diagnoses for every patient and making treatment recommendations based on efficacy instead of convenience can reduce disparities to increase symptom remission and decrease chronic impairment (Medlock 2017)

# Consider how institutional racism manifests in medical education: a substantial amount of White medical students hold false beliefs around biological differences in pain in Black patients, and this racial bias in pain perception negatively affects pain treatment recommendation (Hoffman 2016).

# Listen with intention and evaluate your patients thoughtfully given gaps in medical education; major medical textbooks’ clinical imaging overrepresents light skin tone, underrepresents dark skin tone, and lacks skin tone diversity. This may be a way in which bias enters medical treatment (Louie 2018).

# Recognizing racism as a social determinant of health can change how students learn about an organ system or a disease (Ufomata 2020).

# **Evidence-based practices:**

- Listen for **how your patient may have experienced anti-Black racism**, stigma, and discrimination in their life and specifically in their interactions with the healthcare system.
- Throughout the visit, **give the patient time and space to tell you how racism affects their health.** Avoid interruptions, lead the visit by using silence.
- **Perform active and empathic listening** by using your patients’ words to confirm understanding, asking infrequent, timely, and considered questions, and adopting the ways they refer to themselves.
- Ask **patient-centered questions**, such as **“How does your medical condition affect your life?”** to **avoid monolithic** assumptions about Black patients, such as biased associations between race and socioeconomic status or clinical evaluation of pain.
- As you listen to what your patient tells you, take note of how **individual and institutional biases may influence decision-making** and reflect on it later; remember that medical education underrepresents and misrepresents Black patients in clinical textbooks/images and this may bias diagnosis and treatment.

# **
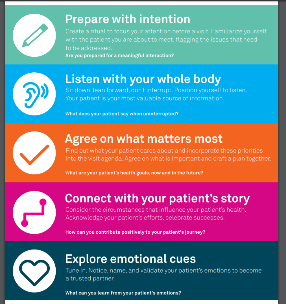
Agree on what matters most**

**Discussion Questions**

- What do you do to ‘Agree on What Matters Most’ with your patients?
- How do you avoid making assumptions about your patient that may affect their healthcare? (e.g., assumptions around medication compliance, experiences with pain, social issues, role of racism in healthcare)
- What do you say to reinforce positive health behaviors in your patient and promote empowerment?

# **Evidence suggests:**

# Black patients who have had more experiences with discrimination with the healthcare system have been shown to trust the healthcare system less compared to white patients (Martin 2013)

# Black patients report lower patient-physician communication quality and satisfaction; less information-giving, partnership building, participatory decision-making, and positive talk; more negative talk; shorter visits; physicians who were more verbally dominant; and worse outcomes on non-verbal communication, respect, and support (Johnson 2004, Cooper 2012, Martin 2013)

# Including the patient in clinical decision-making (e.g., “What do you think caused your problem?" or "What treatment do you think you should receive?") can reduce the likelihood of clinicians making decisions based on preconceptions of specific groups (Kleinman 2003; Medlock 2017)

# Acknowledging the patient’s perspective and including the patient in the conversation can lead to improved patient trust, health outcomes, positive patient-provider communication, and reduced racial disparities (Cuevas 2016)

# When clinicians work with the patient to identify a personal health goal without making assumptions based on race and collectively brainstorm strategies for reaching this goal, patients are more likely to be more involved and committed to working towards the health goal (Drake 2017)

# Implementing standardized protocols for patient interaction and treatment decisions can reduce variations in treatment and outcomes based on implicit biases (Hall 2015, Medlock 2017)

# Physicians should acknowledge racism as a social determinant of health (Paradies 2015) and use this knowledge of social determinants and risk factors to determine what would be appropriate, unbiased referrals to social/community resources (Moffett 2019) Consider appropriate referrals; non-White children are more likely to be evaluated and reported for suspected abuse compared to White children and inappropriately referred to child protective services (Lane 2002)

# **Evidence-based practices:**

- Avoid biased assumptions about patient priorities and the role racism plays in their healthcare, by asking the patient to identify their own goals (**“What are your health goals for today?”**) and share what is most important for them in their healthcare.
- **Partner with your patient** in determining if racism plays a role in their health by asking questions like, **“Is there something else you wanted to discuss today? I want you to know that I am here for you in case you wanted to talk about current events around racism”** to promote patient-driven conversation around race, racism, and historical/current events.
- Perform **shared decision making** and make decisions based on patient presentation (rather than assumptions, for example, around medication non-adherence or social context) to **prevent biased decisions.** Team up with the patient to brainstorm and develop a plan to solve their unique concerns.
- Ensure the patient’s agreement and understanding of their care plan by asking the patient to describe their health goals and treatment plan in their own words to increase their **self-efficacy and confidence** in their ability to manage their health. Use **positive language** (congratulate on successes) to encourage good health behaviors and resilience in the face of racism.
- **Refer appropriately** to interdisciplinary staff (social workers, community health workers, etc.) with the patient’s agreement, understanding historical patterns in inappropriate referrals for Black patients (e.g., increased referrals for child abuse).

**
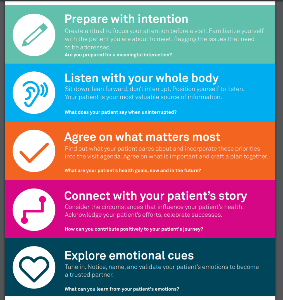
Connect with the patient’s story**

**Discussion Questions**

- What do you do to ‘Connect with the Patient’s Story’?
- Have you reflected on how anti-Black racism impacts the health of your patients?
- What are some examples of racism against your patients that you have witnessed in your clinical education? How has this been addressed? (e.g., assuming child abuse for a Black child with a fracture)
- Are there historical examples of racism in medicine from your institution or geographic region that you know of (e.g., – Henrietta Lacks at Johns Hopkins)?

# **Evidence suggests:**

# Understanding the history of racism in medicine can help combat anti-Black racism by elucidating the historical reasons for low trust in medicine among some Black patients (Thomas 2019)

# Acknowledging a Black patient's past experience with healthcare and perspective can:

- - Initiate dialogue concerning a care plan (Brooks 2016)
  - Identify barriers 'beyond the disease' (Moffett, 2019)
  - Encourage patient advocacy (Dallapiazza, 2018)

# Considering a patient’s perspective and life circumstances can reduce racial biases in clinical treatment (Drwecki 2011, Harmsen 2005)

# When a patient and clinician are racially discordant, acknowledging similarities with patients leads to improved treatment decisions and patient health outcomes (Saha 2008)

# Resident physicians care for a predominantly underserved patient population, and residents’ patients are less likely to receive chronic disease quality of care and preventive cancer screening measures, have higher resource utilization including ED visits and hospital admission, and report lower satisfaction with care (Essien 2019)

# When writing chart notes and presenting the patient’s health story, trainees should eliminate language about race that demonstrates preconceptions about minority racial groups (i.e., “non-compliant,” “poor historian”), which can lead to improved documentation, history taking, and understanding of patients of color through the context of health disparities and historically discriminatory policies (Tsai 2018).

# **Evidence-based practices:**

- Learn about the **historical instances of racism in medicine that Black patients have experienced**, both individually and as a collective shared experience of anti-Black racism. Where might anti-Black racism manifest in your patient’s story?

Acknowledge how racism and other social determinants of health may have created a barrier to healthcare and a healthy lifestyle. Consider asking your patient, **“How have you/ your family/ your community been treated when receiving healthcare?’ or "I know for many of my patients, racial bias is often felt in medical settings or other parts of their lives. I'm wondering if this is true for you, and if you'd be willing to talk a little about that?”**

Be open and curious about possible reasons (sociocultural, biological, psychological, etc.) for a **patient’s individual beliefs** and actions and appreciate that a **patient's racial identity may or may not contribute** to these.

- Acknowledge that, as a trainee, your relationship with a patient may be short-term and how that may affect their care; **where do you fit within a patient’s life story?** How does your short-term relationship affect your ability to provide equitable care?
- Appreciate that you may have more time to spend with a patient as a trainee. Ensure high quality continuity of care by asking, **“Is there anything specific that we've discussed today that I can pass on to my attending to improve your healthcare beyond this encounter?”**

As you connect with your patient’s story, carefully choose how you write about and present that story to your attendings. **Do you acknowledge the complicated social factors that influence their health status? Is information about race relevant to the case?** Avoid including information about race that may bias a listener or reader.

**
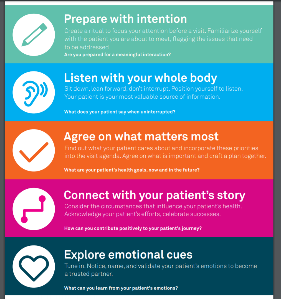
Explore emotional cues**

**Discussion Questions**

- What do you do to ‘Explore Emotional Cues’ with your patient?
- How do you name emotions that you observe in the moment when it relates to racial trauma?
- What elements of trauma informed care can you apply when addressing anti-Black racism and healthcare?
- What do you do to promote trust, especially in the context of historical examples in medicine causing distrust in the Black community? (e.g., Tuskegee Syphilis Experiment)

# **Evidence suggests:**

# Clinicians limit discussion of emotional issues more with Black patients and provide Black patients with fewer passive responses that permit emotional talk (Park 2020)

# Clinician perception of patient emotions is associated with greater patient satisfaction, appointment adherence, and learning of conveyed information (Hall, 2011, Weng, 2011) While individuals’ emotional sensitivity may vary widely, research shows that patients appreciate physician attempts to elicit and identify their emotional cues, even when the clinician is mistaken (Blanch-Hartigan 2013)

# When witnessing racial trauma in clinical interactions, use the INTERRUPT framework (inquire, nonthreatening, take responsibility, empower, reframe, redirect, use impact questions, paraphrase, teach) to explore emotional cues by asking questions, re-directing, and teaching from your own experience as a trainee (DallaPiazza 2018)

| I—Inquire: Leverage curiosity. “I'm curious, what makes you think/say that?” |
| --- |
| N—Nonthreatening: Convey the message with respect. Separate the person from the action or behavior. “Some may consider that statement to be offensive.” Communicate preferences rather than demands. “It would be helpful to me if….” |
| T—Take responsibility: If you need to reconsider a statement/action, acknowledge and apologize. Address microaggressions, and revisit them if they were initially unaddressed. |
| E—Empower: Ask questions that will make a difference. “What could you/we do differently?” |
| R—Reframe: “Have you ever thought about it like this?” |
| R—Redirect: helpful when individuals are put on the spot to speak for their identity group. “Let's shift the conversation….” |
| U—Use impact questions: “What would happen if you considered the impact on … ?” |
| P—Paraphrase: making what is invisible (unconscious bias) visible. “It sounds like you think….” |
| T—Teach by using “I” phrases: Speak from your own experience. “I felt x when y happened, and it impacted me because….” |

# Pay attention to building trust, empathy, and self-efficacy into your relationships with minority patients (DallaPiazza 2018) and participate in education in interpretation of nonverbal behavior and accommodate norms of local patient populations (Lorie 2017)

# Trauma-informed care can help trainees provide quality care (Elisseou 2019) and understanding the concept of embodiment, in the context of racial trauma, can help trainees understand how the lived experience of racism manifests in patients physically in their body (Verghese 2018)

# **Evidenced-based practices:**

- **Reflect, validate, and confirm** your perceptions of a patient’s emotions around racial trauma and racism in medicine (e.g. **“That sounds very difficult”** or **“I can see that this is affecting you deeply”**)
- **Read a patient’s verbal and non-verbal emotional cues**, e.g. changes in patient tone of voice, facial expressions, and body language. If you sense the patient was offended by something said, ask, **"I see that you're upset by something just now, is that correct?"**
- **Recognize racial trauma and name racism** when it comes up in the moment of the clinical interaction (e.g. **“I am so sorry that we failed to recognize your pain sooner and I realize that racism may play a role in this”**)
- Apply elements of **trauma informed care** especially considering racial trauma so that the patient's experiences are recognized within the delivery of the visit (e.g. use the patient’s language and self-identified race and always ask for permission before asking sensitive questions and performing a physical exam)

**Goal Setting and Action Plan to Adopt a New Practice**

*General reflection*

- - What do you want to try that you heard today?

*Create realistic goals with concrete objectives*

- - What barriers and gaps have you identified in your medical education that may perpetuate racial bias? What are you going to specifically do to promote racial justice for your patients in your medical education?
  - What do you need to achieve your goals?
  - What might be challenging/what are some barriers to achieving your goals?

*Questions for follow-up discussion groups*

- - How has this been going for you? What have you noticed (things you already do, things that you now pay more attention to, changes you have made as a result of keeping presence/racial justice in mind)?
  - How do you ensure that generalizations are not being made when practicing anti-racism in your clinical interactions with Black patients?
  - What do you do to act as an upstander in the face of anti-Black racism in your clinical interactions and education?
  - What kind of anti-racism skills do you feel like you need in your medical education to promote racial justice when caring for your patients? (e.g., how can schools address the gaps noted in the racial justice report cards from #WhiteCoatsforBlackLives?)
  - What are the biggest barriers (systemic and individual) to use these anti-racism practices?
  - What can you do to ensure that advancing racial justice in your clinical encounters is a life-long commitment rather than a short-term goal?


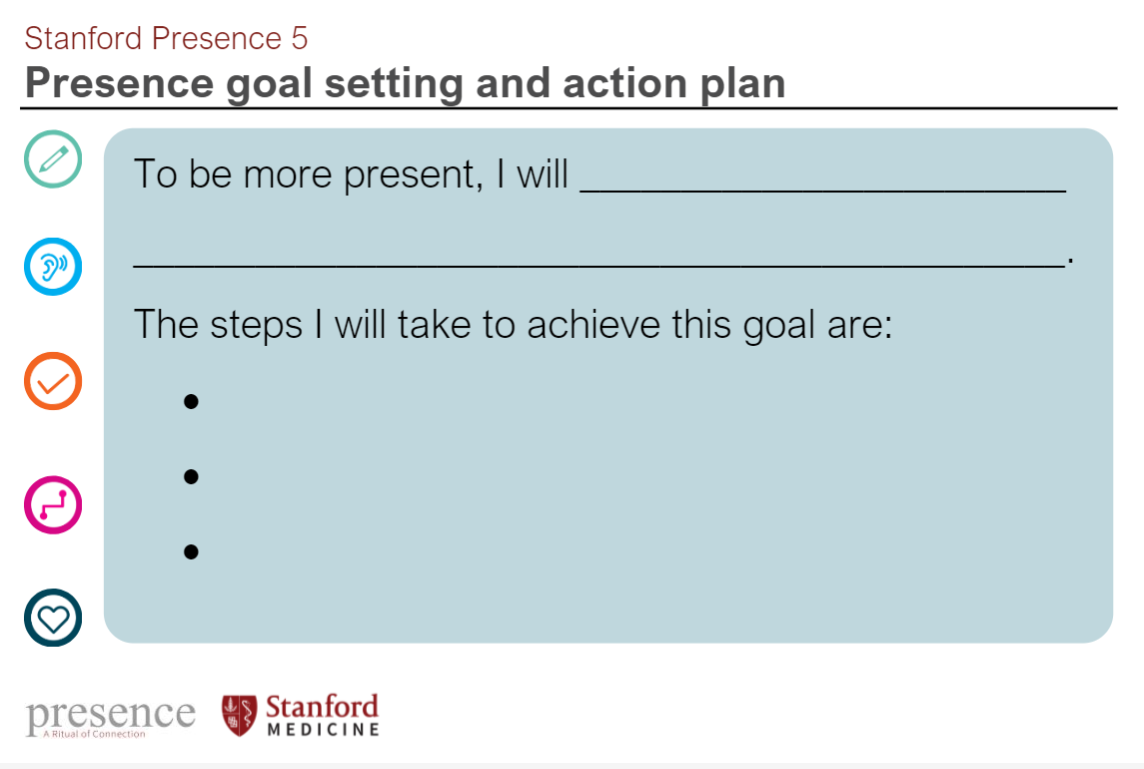


**
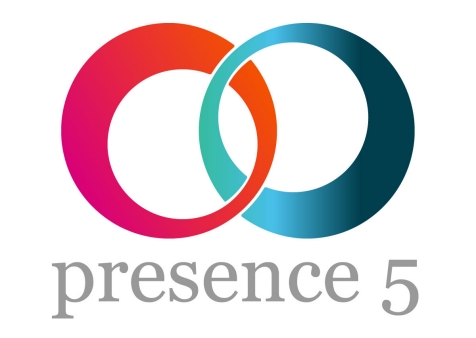
**
