# Supplementary material for: Presence 5 for Racial Justice Workshop: Fostering Dialogue Across Medical Education to Disrupt Anti-Black Racism in Clinical Encounters
Source: MedEdPORTAL. 2022 Feb 10;18:11227. doi: 10.15766/mep_2374-8265.11227 (PMC8828658; doi:10.15766/mep_2374-8265.11227)
Supplement: Supplementary file 1 — Presence 5 for Racial Justice Guide.docxIntroductory Didactic.pptxParticipant Resources.docxSurvey.docx [file mep_2374-8265.11227-s001.zip › B. Introductory Didactic.pptx]

## Slide 1
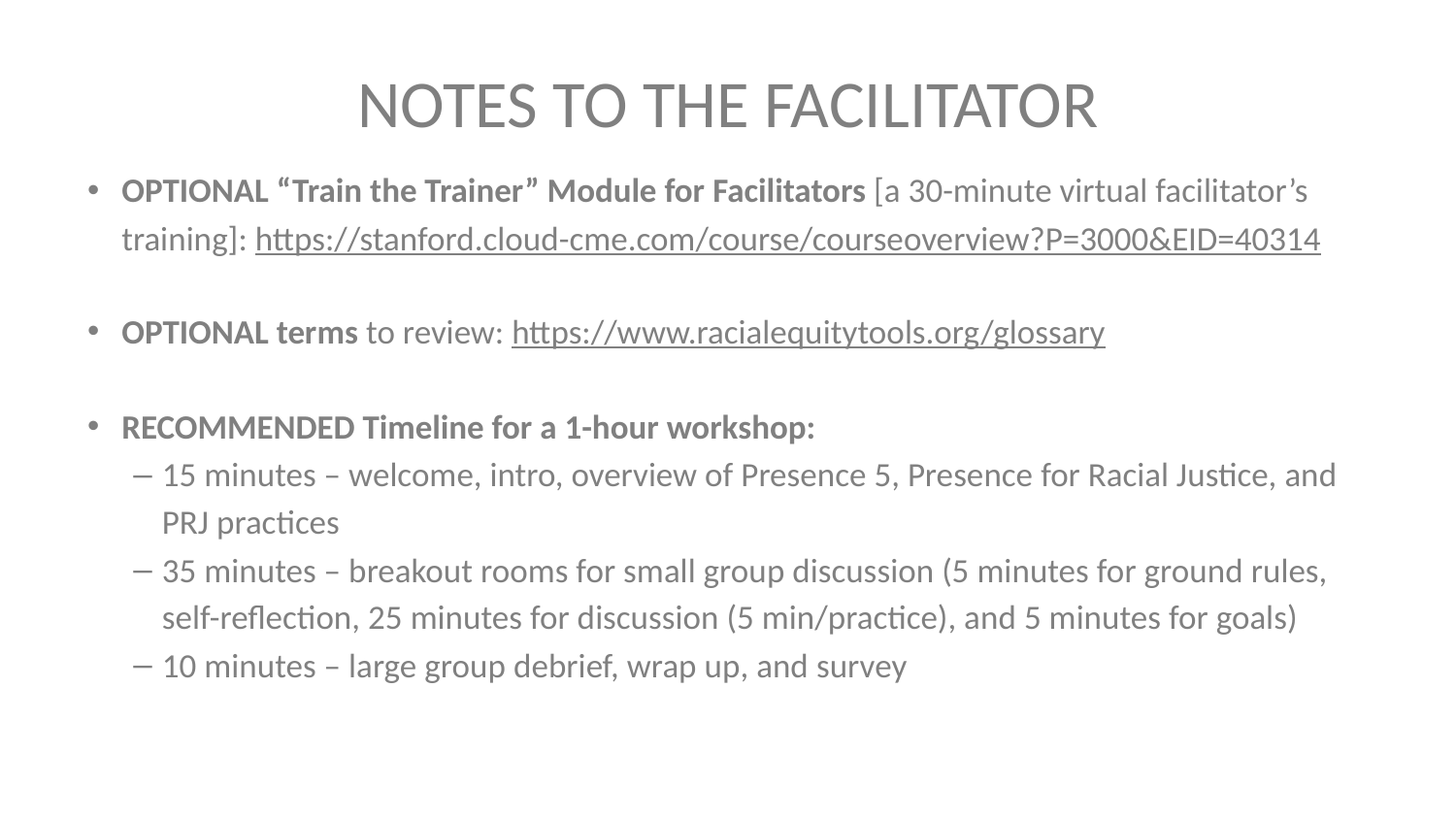

# NOTES TO THE FACILITATOR
OPTIONAL “Train the Trainer” Module for Facilitators [a 30-minute virtual facilitator’s training]: https://stanford.cloud-cme.com/course/courseoverview?P=3000&EID=40314
OPTIONAL terms to review: https://www.racialequitytools.org/glossary
RECOMMENDED Timeline for a 1-hour workshop:
15 minutes – welcome, intro, overview of Presence 5, Presence for Racial Justice, and PRJ practices
35 minutes – breakout rooms for small group discussion (5 minutes for ground rules, self-reflection, 25 minutes for discussion (5 min/practice), and 5 minutes for goals)
10 minutes – large group debrief, wrap up, and survey

## Slide 2
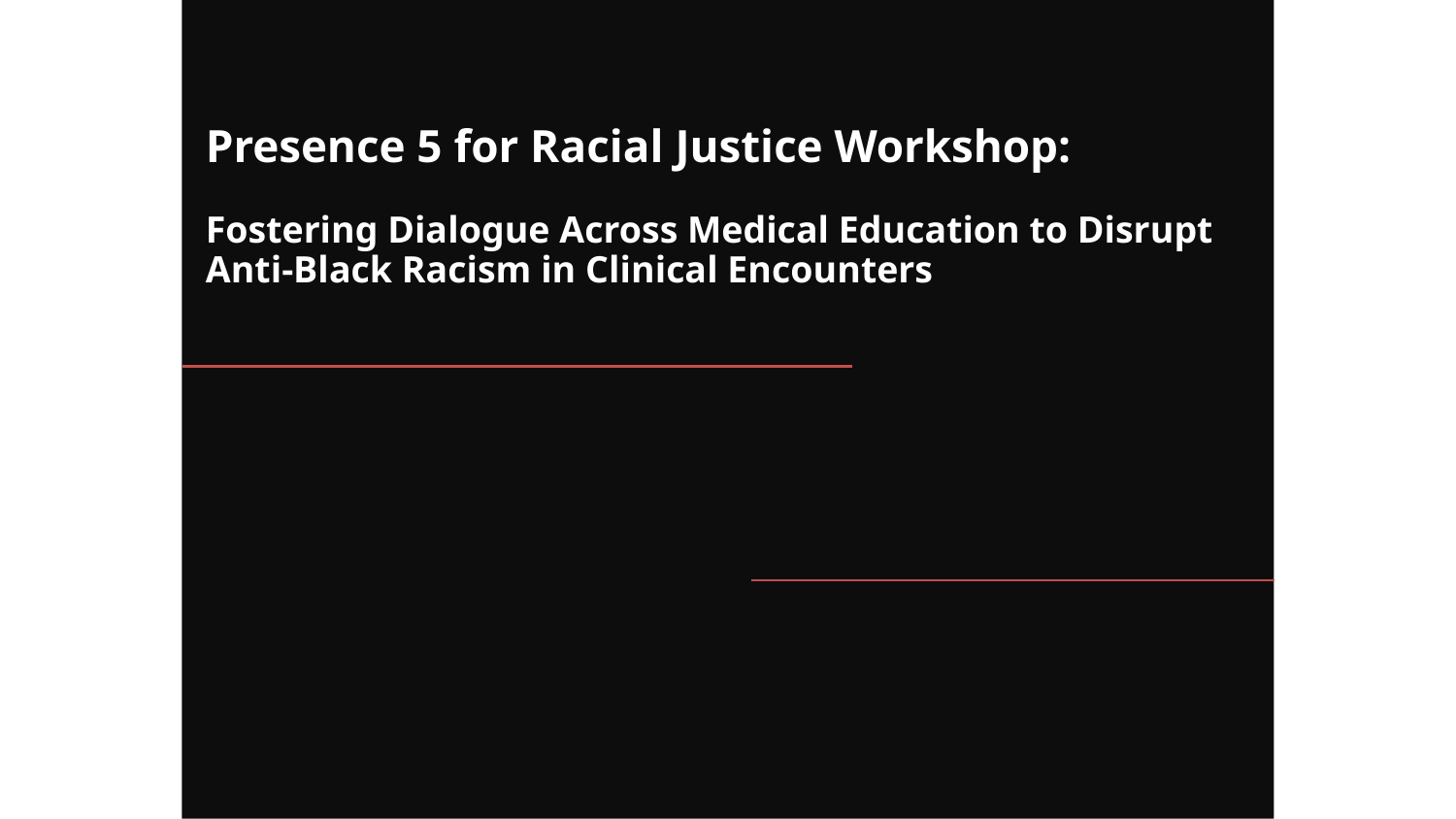

# Presence 5 for Racial Justice Workshop: Fostering Dialogue Across Medical Education to Disrupt Anti-Black Racism in Clinical Encounters

## Slide 3
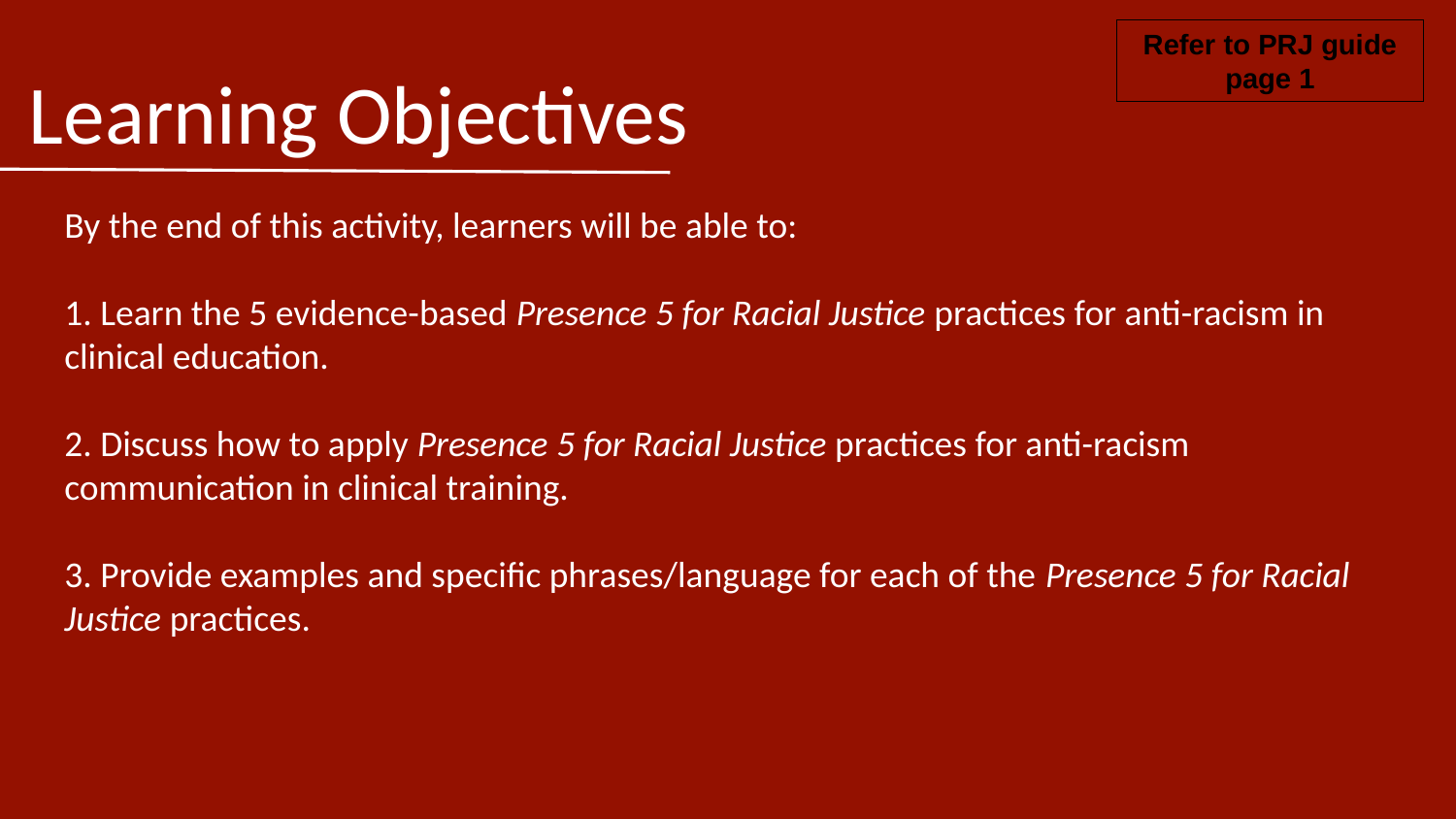

# Learning Objectives
Refer to PRJ guide page 1
By the end of this activity, learners will be able to:
1. Learn the 5 evidence-based Presence 5 for Racial Justice practices for anti-racism in clinical education.
2. Discuss how to apply Presence 5 for Racial Justice practices for anti-racism communication in clinical training.
3. Provide examples and specific phrases/language for each of the Presence 5 for Racial Justice practices.

## Slide 4
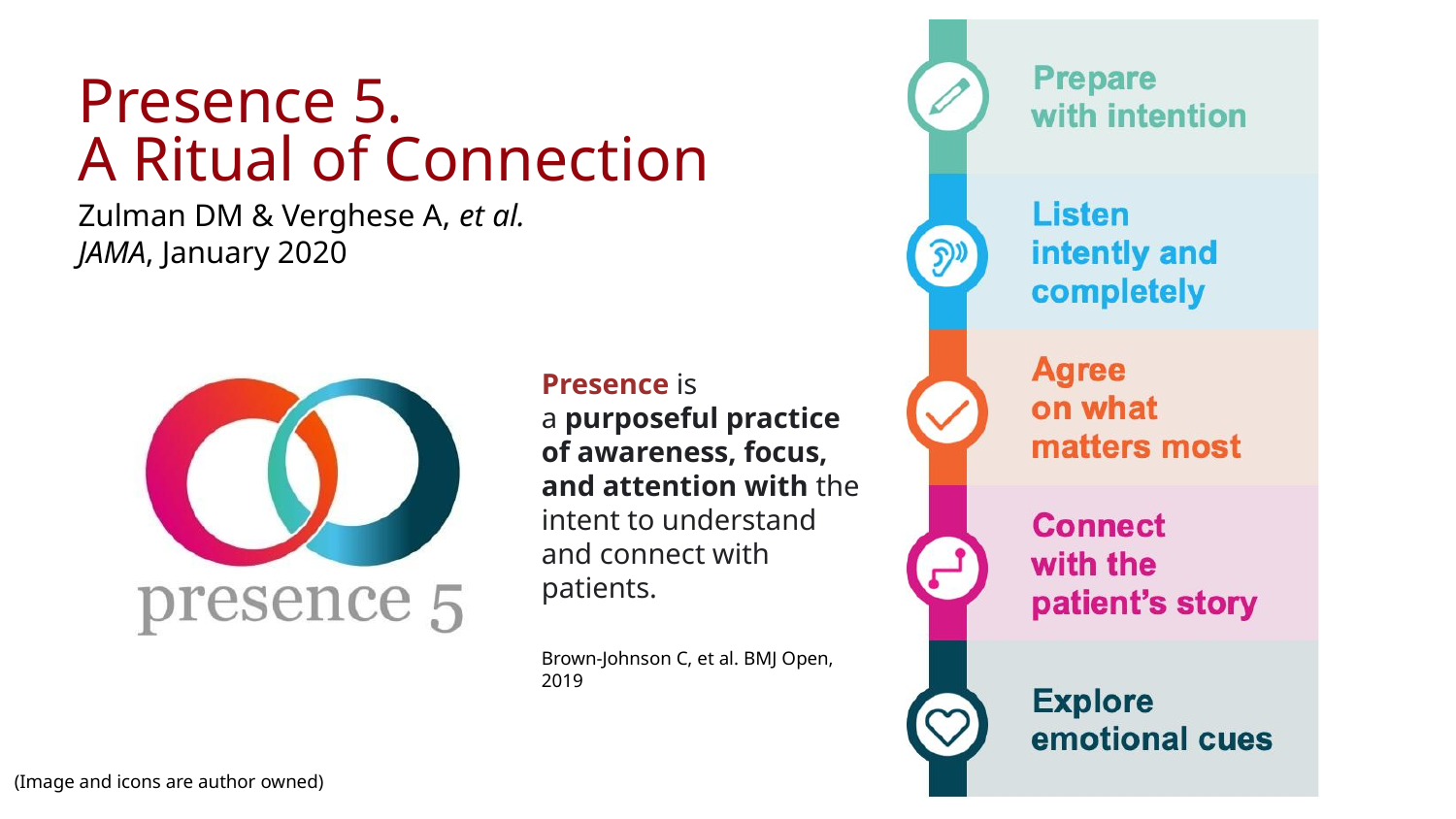

Presence 5.
A Ritual of Connection
Zulman DM & Verghese A, et al.
JAMA, January 2020
Presence is a purposeful practice of awareness, focus, and attention with the intent to understand and connect with patients.
Brown-Johnson C, et al. BMJ Open, 2019
(Image and icons are author owned)

## Slide 5
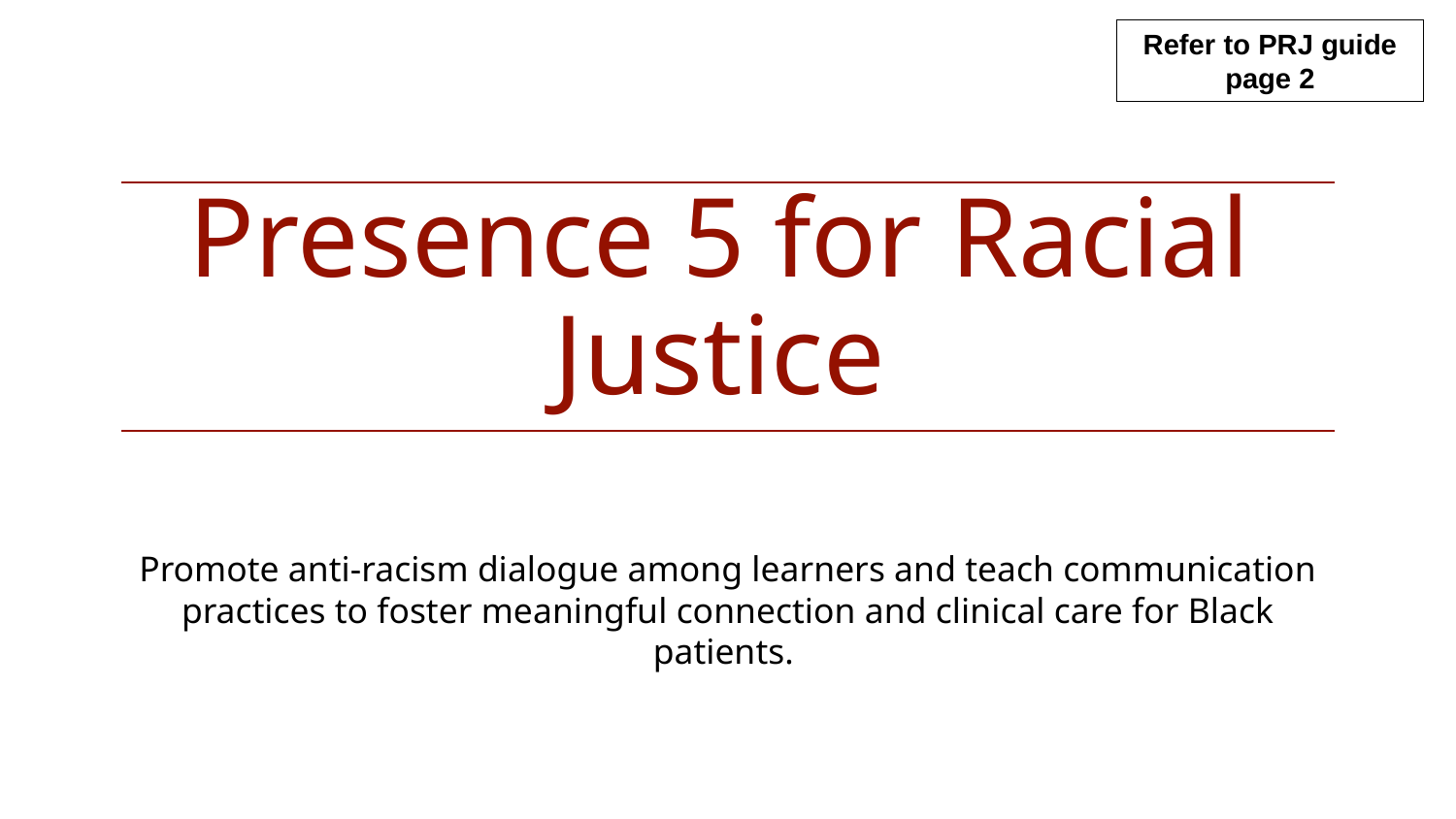

Refer to PRJ guide page 2
# Presence 5 for Racial Justice
Promote anti-racism dialogue among learners and teach communication practices to foster meaningful connection and clinical care for Black patients.

## Slide 6
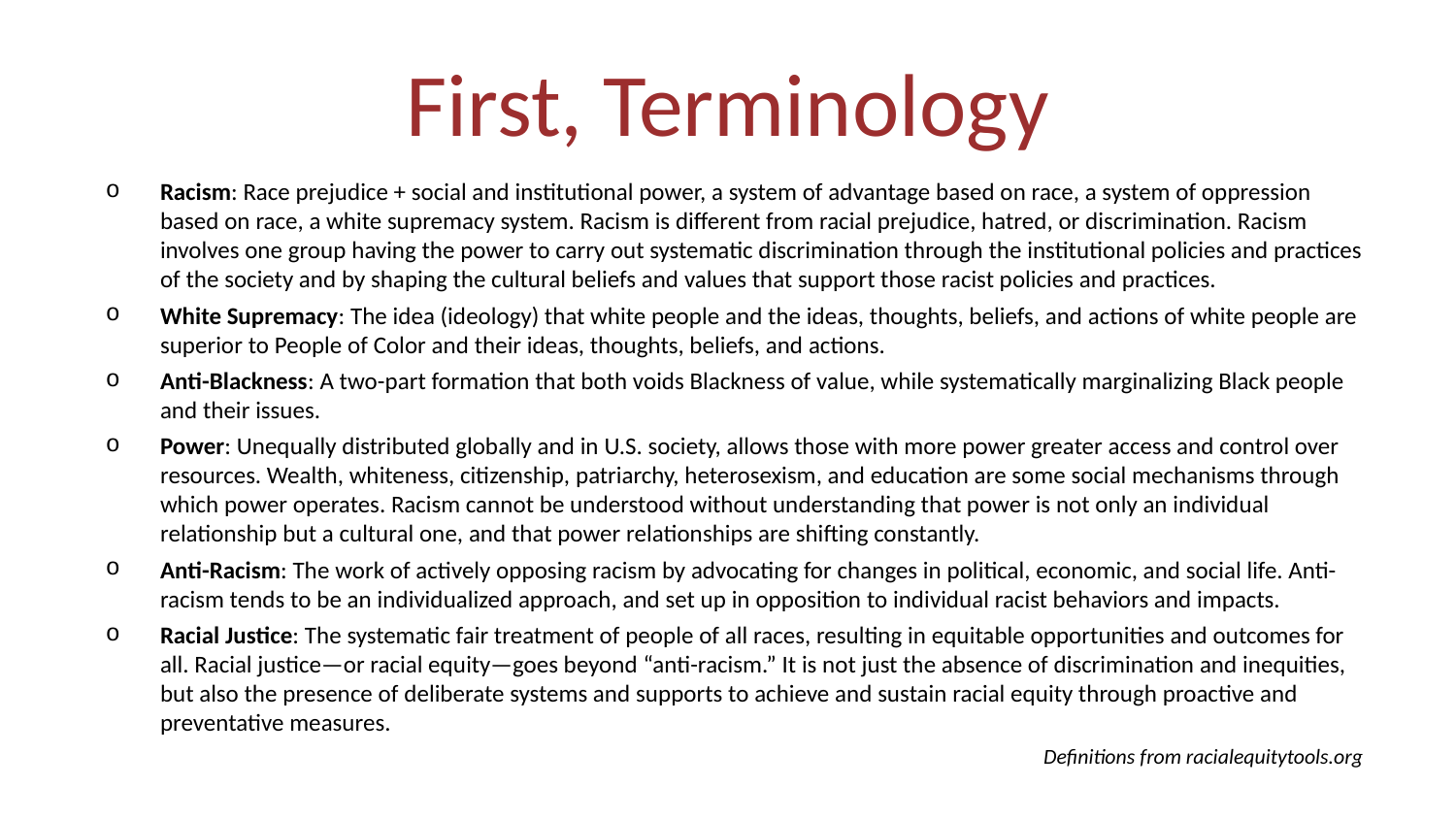

# First, Terminology
Racism: Race prejudice + social and institutional power, a system of advantage based on race, a system of oppression based on race, a white supremacy system. Racism is different from racial prejudice, hatred, or discrimination. Racism involves one group having the power to carry out systematic discrimination through the institutional policies and practices of the society and by shaping the cultural beliefs and values that support those racist policies and practices.
White Supremacy: The idea (ideology) that white people and the ideas, thoughts, beliefs, and actions of white people are superior to People of Color and their ideas, thoughts, beliefs, and actions.
Anti-Blackness: A two-part formation that both voids Blackness of value, while systematically marginalizing Black people and their issues.
Power: Unequally distributed globally and in U.S. society, allows those with more power greater access and control over resources. Wealth, whiteness, citizenship, patriarchy, heterosexism, and education are some social mechanisms through which power operates. Racism cannot be understood without understanding that power is not only an individual relationship but a cultural one, and that power relationships are shifting constantly.
Anti-Racism: The work of actively opposing racism by advocating for changes in political, economic, and social life. Anti-racism tends to be an individualized approach, and set up in opposition to individual racist behaviors and impacts.
Racial Justice: The systematic fair treatment of people of all races, resulting in equitable opportunities and outcomes for all. Racial justice—or racial equity—goes beyond “anti-racism.” It is not just the absence of discrimination and inequities, but also the presence of deliberate systems and supports to achieve and sustain racial equity through proactive and preventative measures.
Definitions from racialequitytools.org

## Slide 7
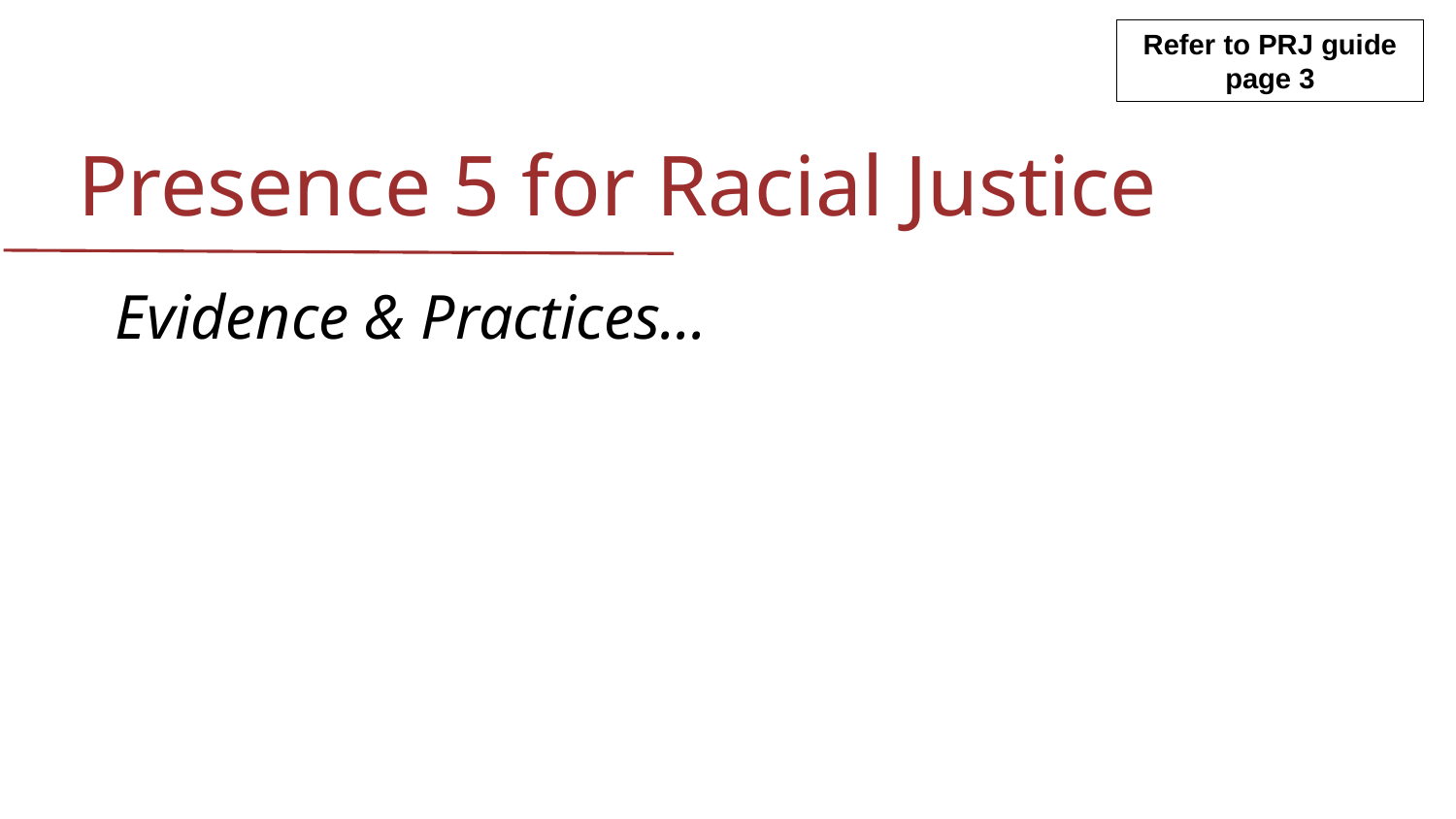

Refer to PRJ guide page 3
# Presence 5 for Racial Justice
Evidence & Practices...

## Slide 8
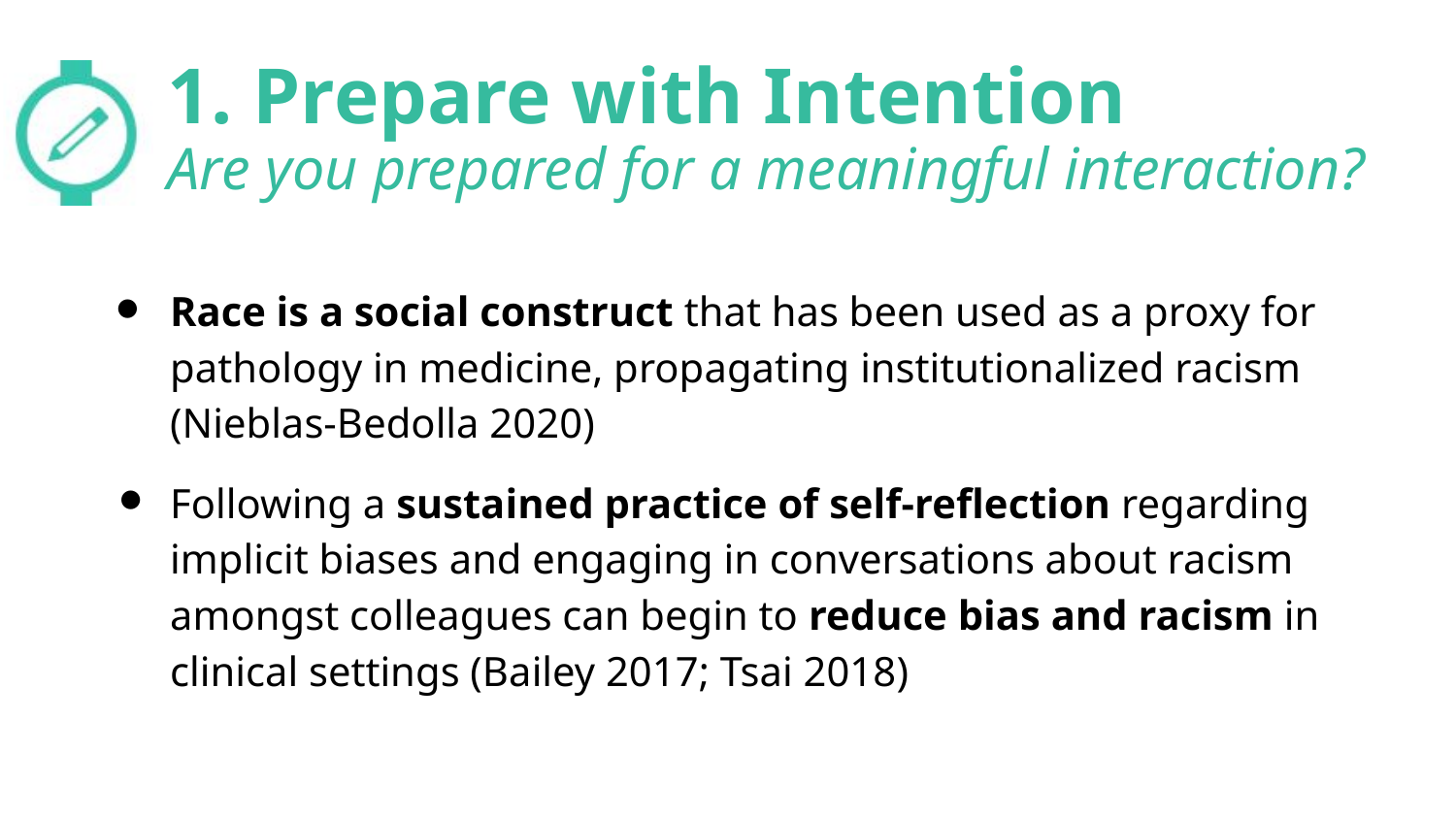

# 1. Prepare with Intention Are you prepared for a meaningful interaction?
Race is a social construct that has been used as a proxy for pathology in medicine, propagating institutionalized racism (Nieblas-Bedolla 2020)
Following a sustained practice of self-reflection regarding implicit biases and engaging in conversations about racism amongst colleagues can begin to reduce bias and racism in clinical settings (Bailey 2017; Tsai 2018)

## Slide 9
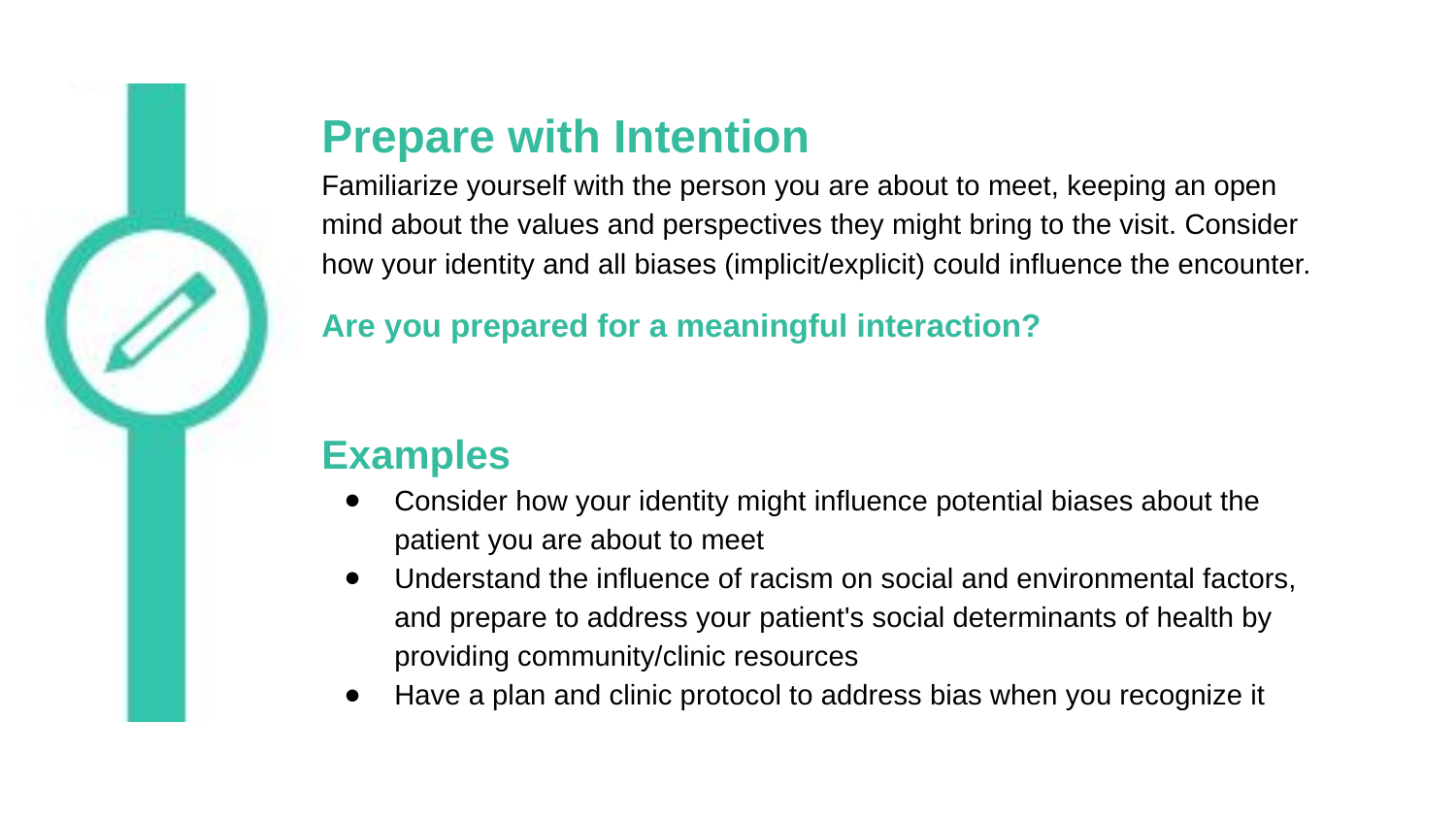

Prepare with Intention
Familiarize yourself with the person you are about to meet, keeping an open mind about the values and perspectives they might bring to the visit. Consider how your identity and all biases (implicit/explicit) could influence the encounter.
Are you prepared for a meaningful interaction?
Examples
Consider how your identity might influence potential biases about the patient you are about to meet
Understand the influence of racism on social and environmental factors, and prepare to address your patient's social determinants of health by providing community/clinic resources
Have a plan and clinic protocol to address bias when you recognize it

## Slide 10
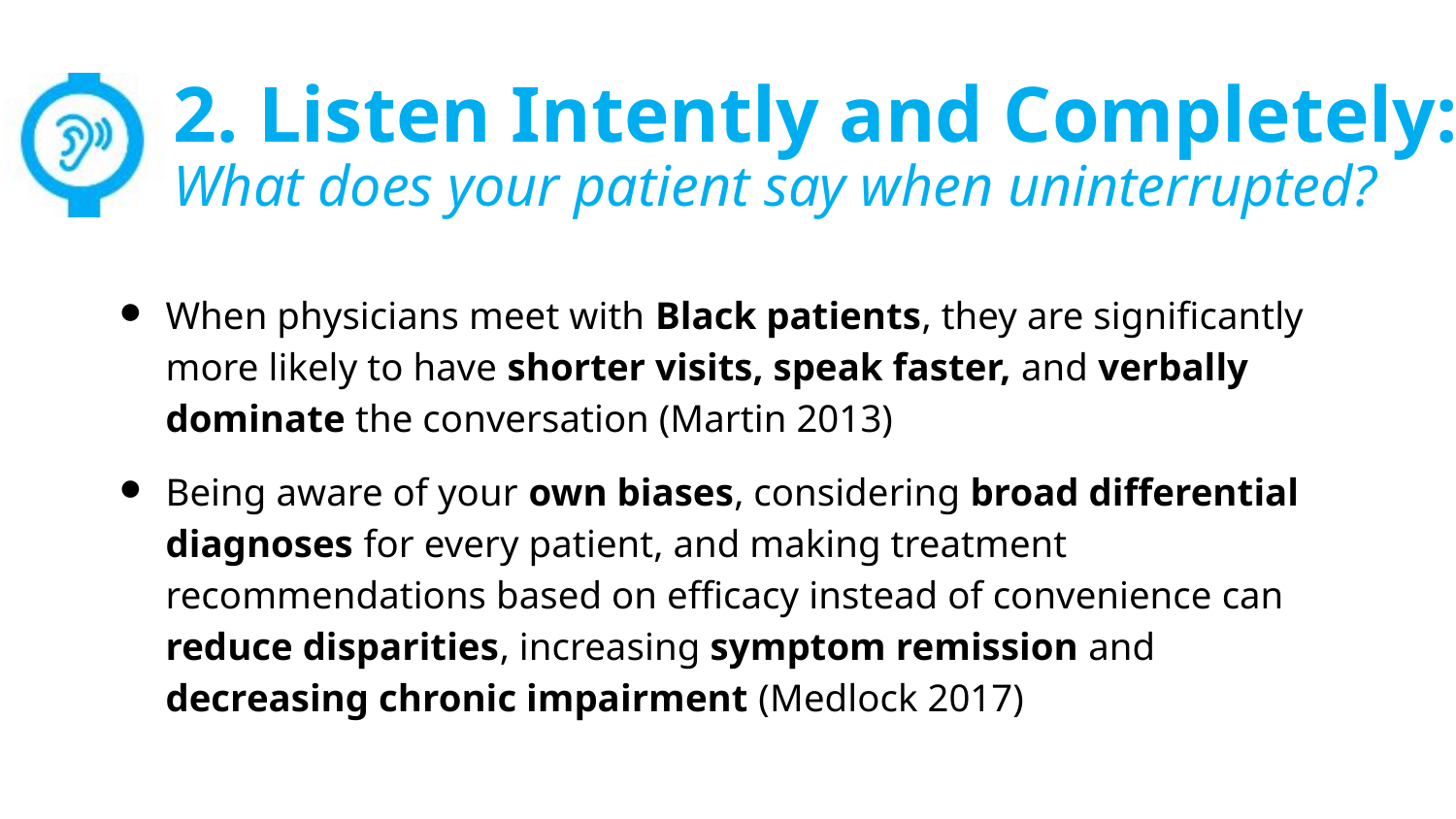

# 2. Listen Intently and Completely:
What does your patient say when uninterrupted?
When physicians meet with Black patients, they are significantly more likely to have shorter visits, speak faster, and verbally dominate the conversation (Martin 2013)
Being aware of your own biases, considering broad differential diagnoses for every patient, and making treatment recommendations based on efficacy instead of convenience can reduce disparities, increasing symptom remission and decreasing chronic impairment (Medlock 2017)

## Slide 11
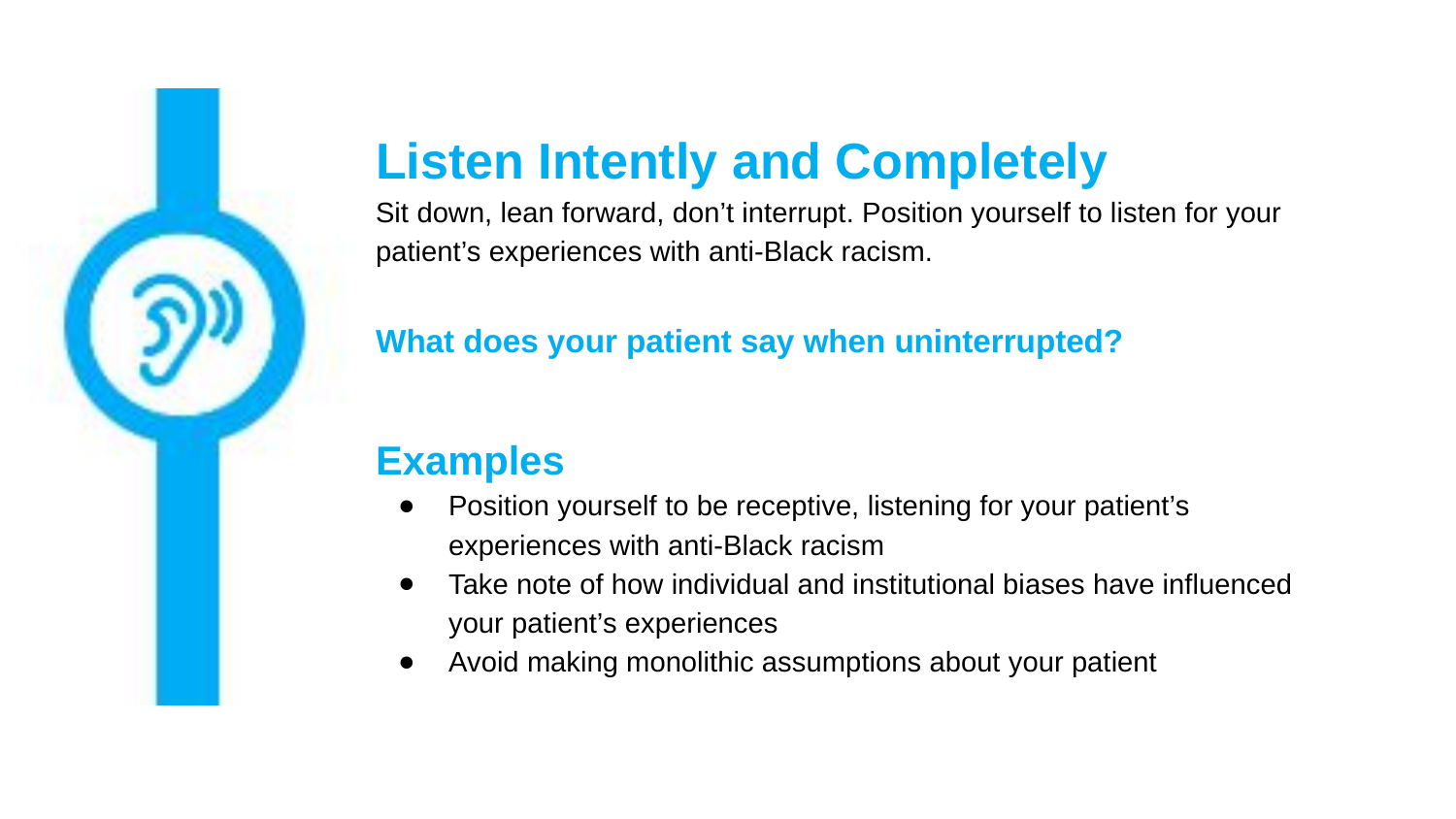

Listen Intently and Completely
Sit down, lean forward, don’t interrupt. Position yourself to listen for your patient’s experiences with anti-Black racism.
What does your patient say when uninterrupted?
Examples
Position yourself to be receptive, listening for your patient’s experiences with anti-Black racism
Take note of how individual and institutional biases have influenced your patient’s experiences
Avoid making monolithic assumptions about your patient

## Slide 12
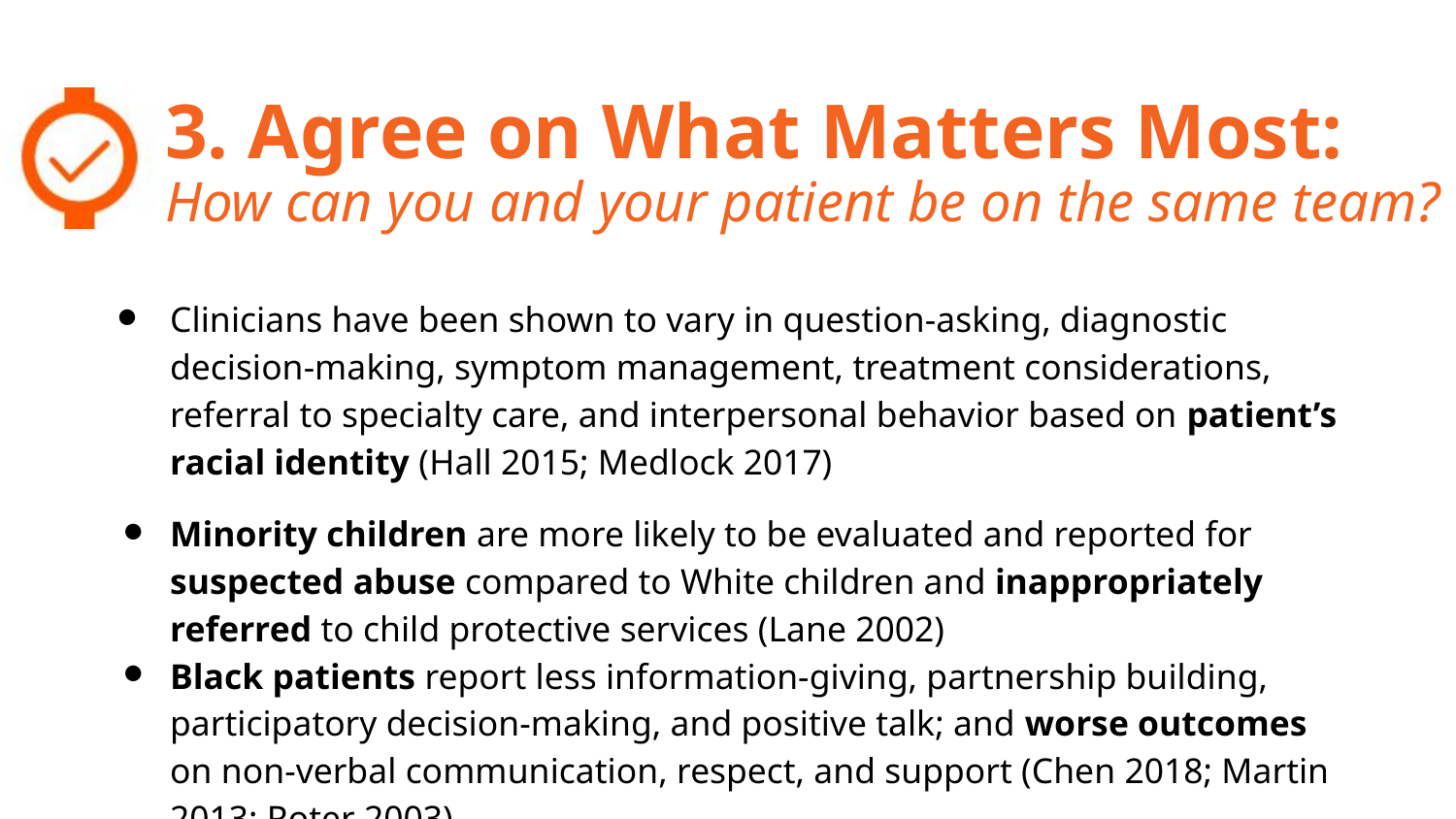

# 3. Agree on What Matters Most:
How can you and your patient be on the same team?
Clinicians have been shown to vary in question-asking, diagnostic decision-making, symptom management, treatment considerations, referral to specialty care, and interpersonal behavior based on patient’s racial identity (Hall 2015; Medlock 2017)
Minority children are more likely to be evaluated and reported for suspected abuse compared to White children and inappropriately referred to child protective services (Lane 2002)
Black patients report less information-giving, partnership building, participatory decision-making, and positive talk; and worse outcomes on non-verbal communication, respect, and support (Chen 2018; Martin 2013; Roter 2003)

## Slide 13
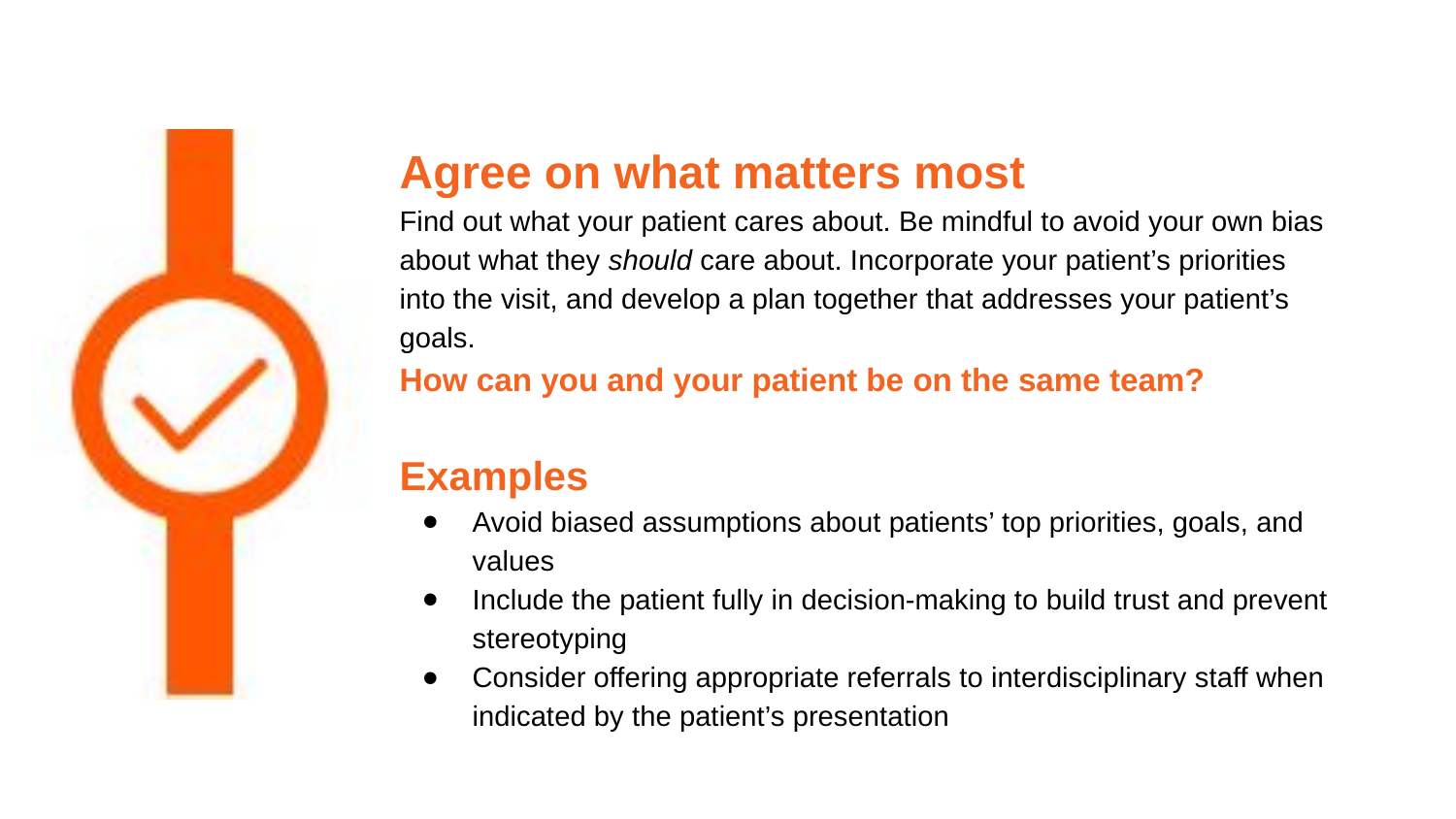

Agree on what matters most
Find out what your patient cares about. Be mindful to avoid your own bias about what they should care about. Incorporate your patient’s priorities into the visit, and develop a plan together that addresses your patient’s goals.
How can you and your patient be on the same team?
Examples
Avoid biased assumptions about patients’ top priorities, goals, and values
Include the patient fully in decision-making to build trust and prevent stereotyping
Consider offering appropriate referrals to interdisciplinary staff when indicated by the patient’s presentation

## Slide 14
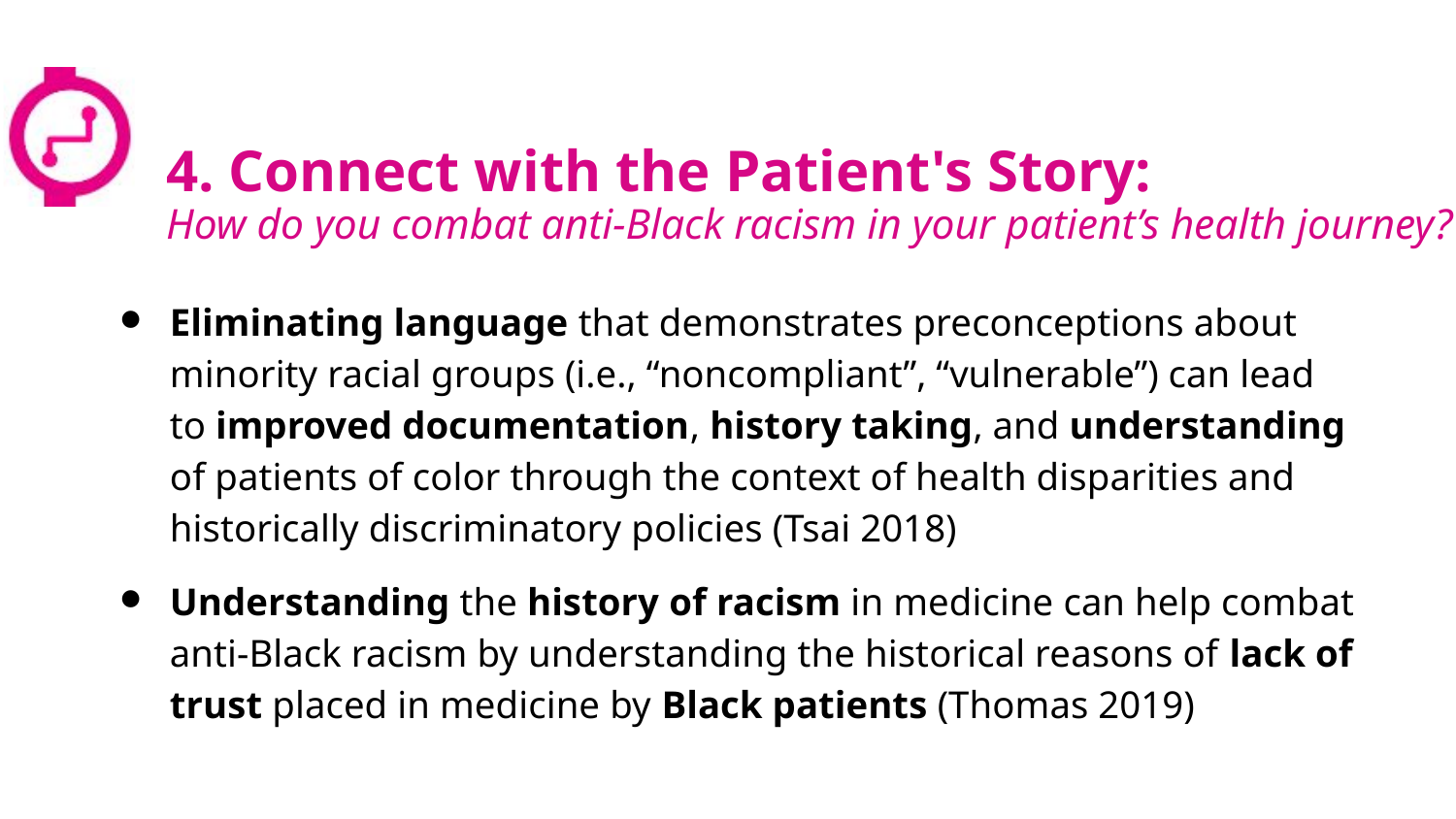

# 4. Connect with the Patient's Story:
How do you combat anti-Black racism in your patient’s health journey?
Eliminating language that demonstrates preconceptions about minority racial groups (i.e., “noncompliant”, “vulnerable”) can lead to improved documentation, history taking, and understanding of patients of color through the context of health disparities and historically discriminatory policies (Tsai 2018)
Understanding the history of racism in medicine can help combat anti-Black racism by understanding the historical reasons of lack of trust placed in medicine by Black patients (Thomas 2019)

## Slide 15
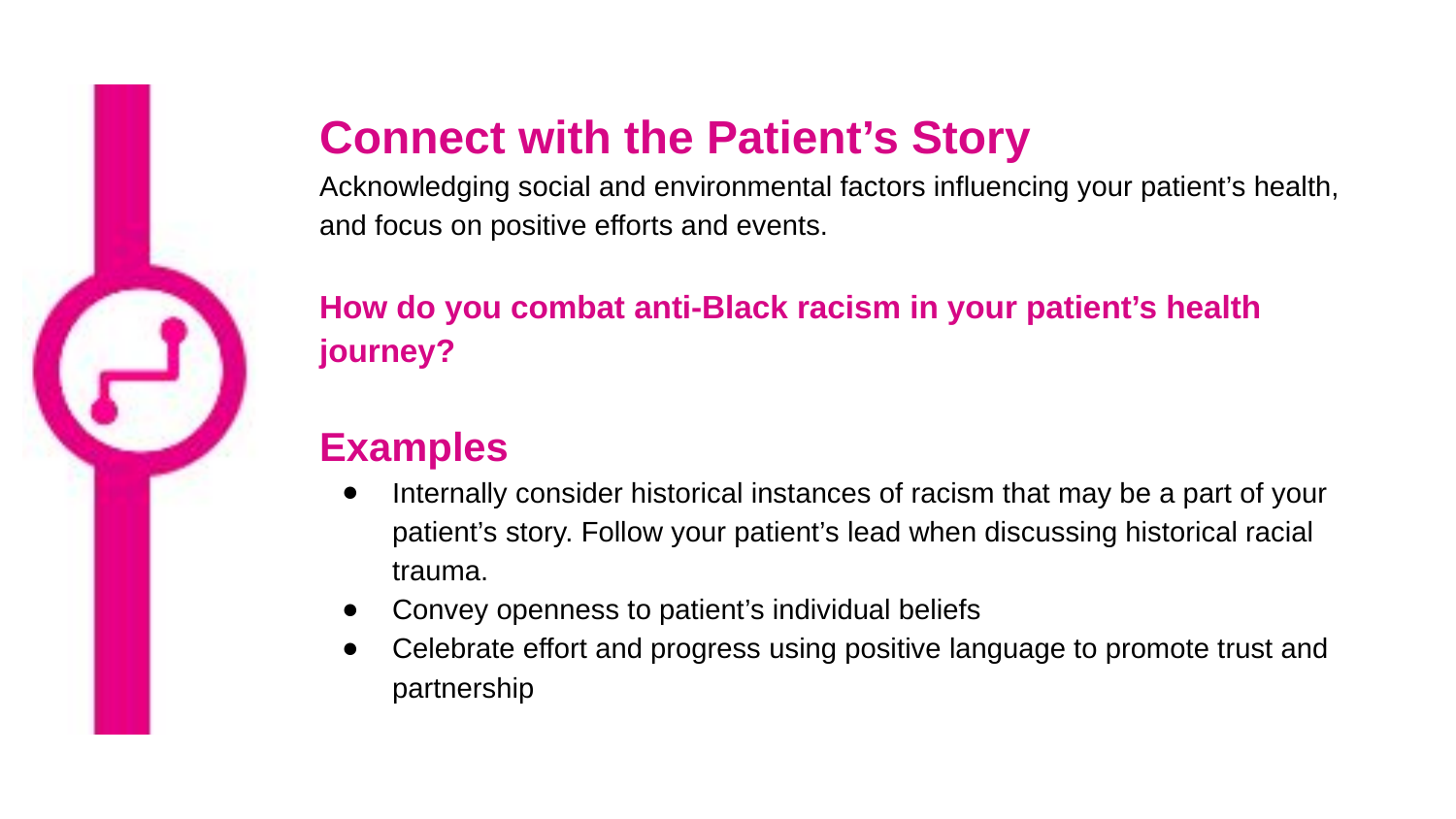

Connect with the Patient’s Story
Acknowledging social and environmental factors influencing your patient’s health, and focus on positive efforts and events.
How do you combat anti-Black racism in your patient’s health journey?
Examples
Internally consider historical instances of racism that may be a part of your patient’s story. Follow your patient’s lead when discussing historical racial trauma.
Convey openness to patient’s individual beliefs
Celebrate effort and progress using positive language to promote trust and partnership

## Slide 16
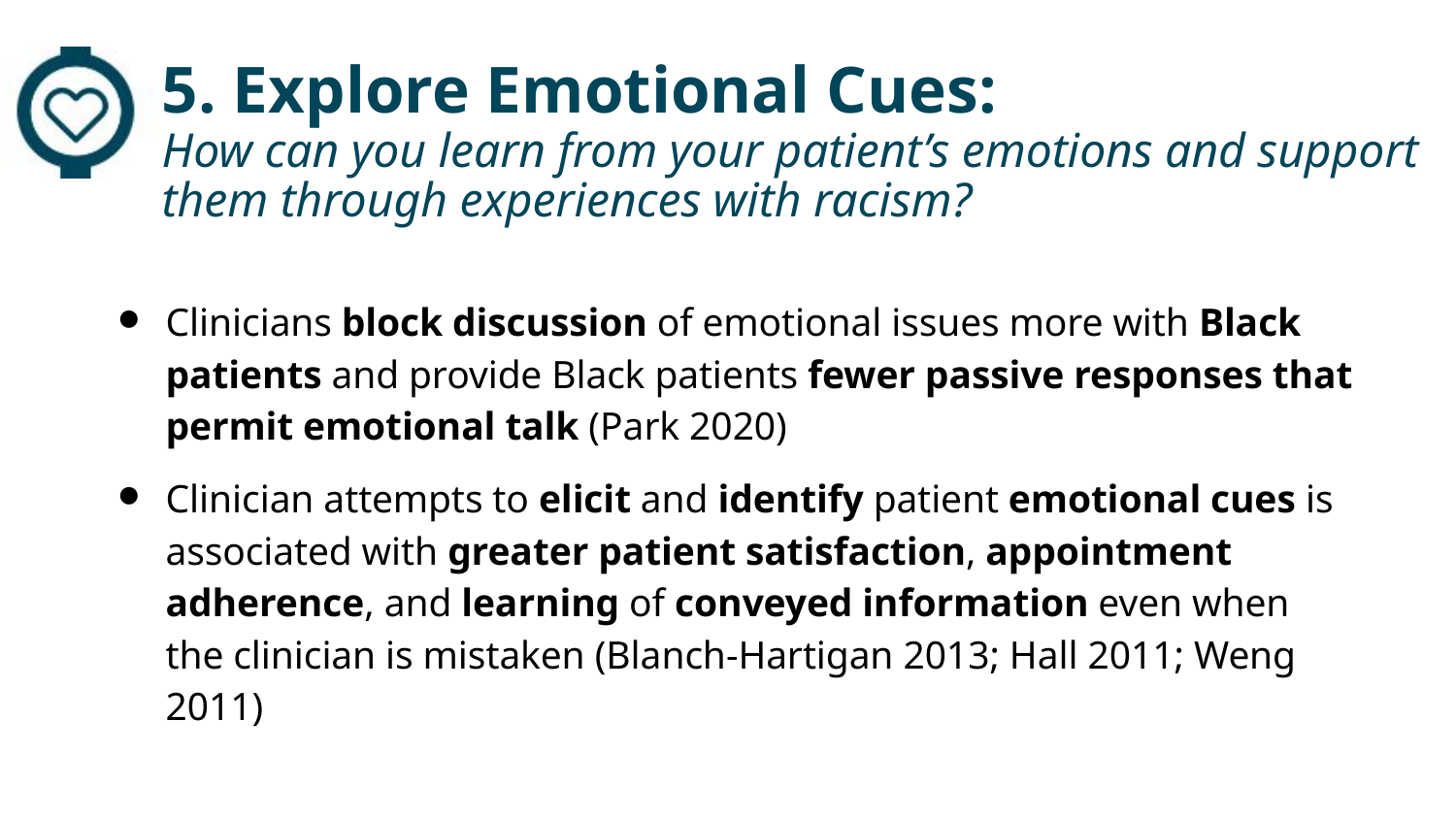

# 5. Explore Emotional Cues:
How can you learn from your patient’s emotions and support them through experiences with racism?
Clinicians block discussion of emotional issues more with Black patients and provide Black patients fewer passive responses that permit emotional talk (Park 2020)
Clinician attempts to elicit and identify patient emotional cues is associated with greater patient satisfaction, appointment adherence, and learning of conveyed information even when the clinician is mistaken (Blanch-Hartigan 2013; Hall 2011; Weng 2011)

## Slide 17
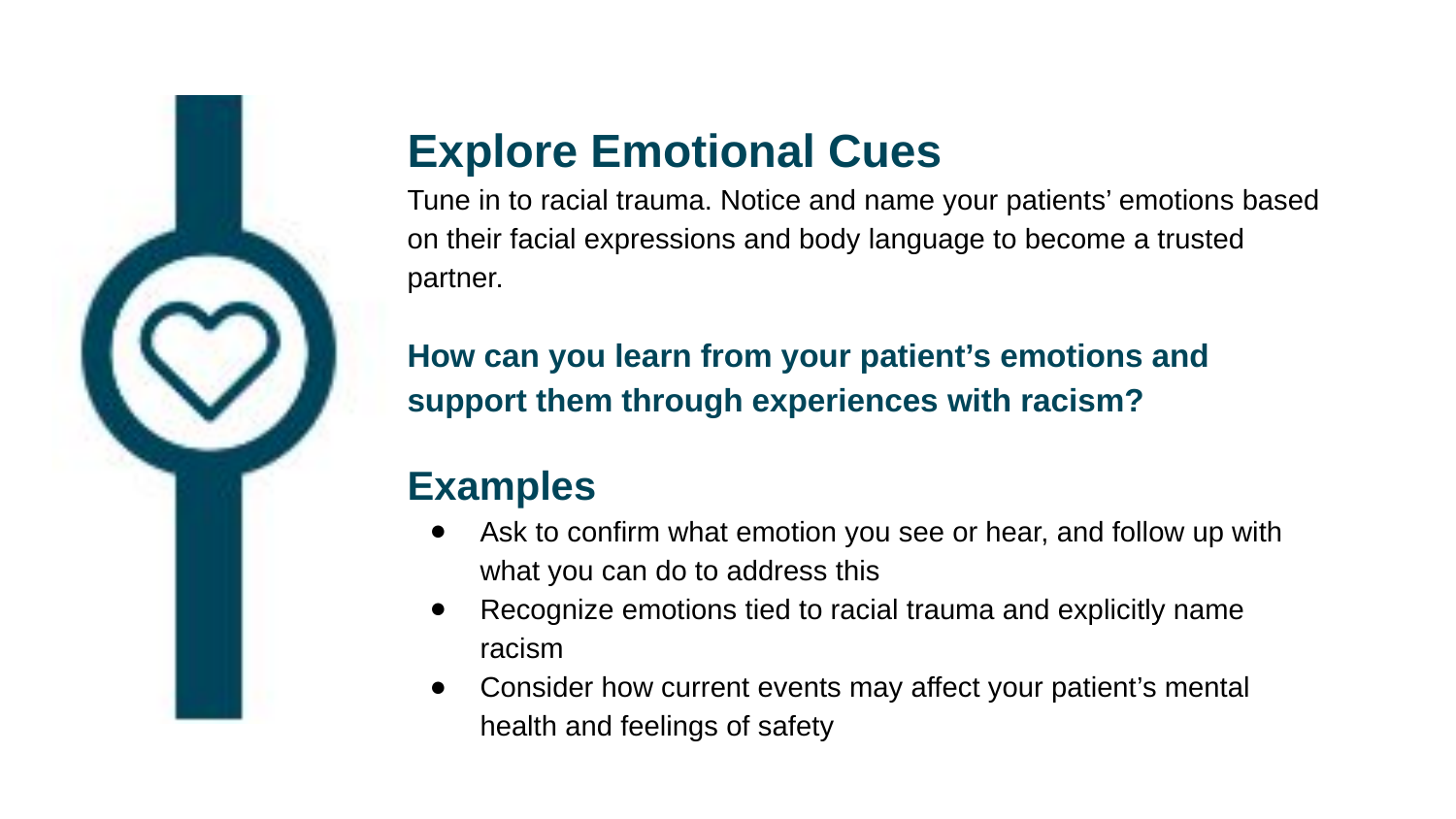

Explore Emotional Cues
Tune in to racial trauma. Notice and name your patients’ emotions based on their facial expressions and body language to become a trusted partner.
How can you learn from your patient’s emotions and support them through experiences with racism?
Examples
Ask to confirm what emotion you see or hear, and follow up with what you can do to address this
Recognize emotions tied to racial trauma and explicitly name racism
Consider how current events may affect your patient’s mental health and feelings of safety

## Slide 18
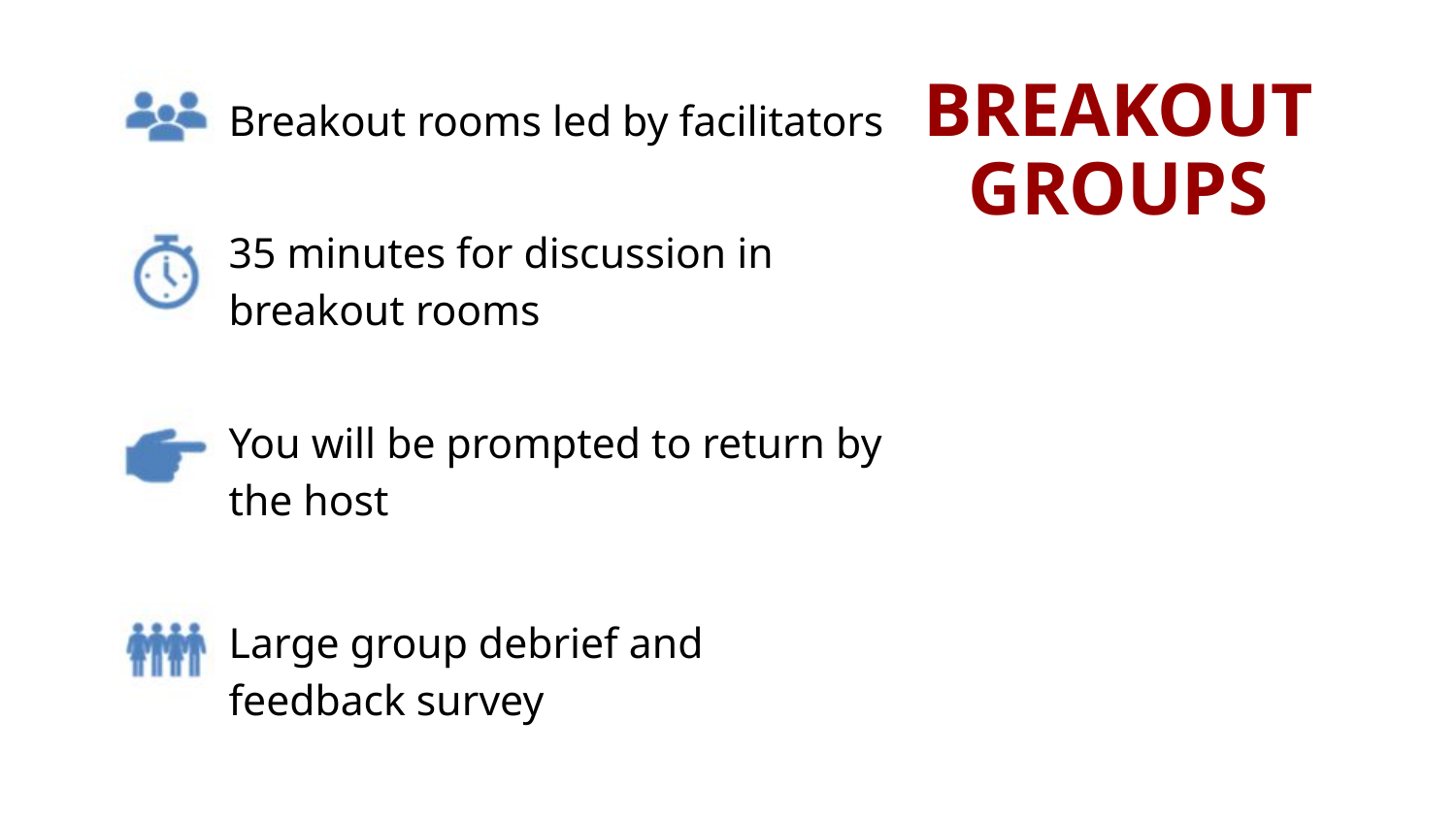

Breakout rooms led by facilitators
35 minutes for discussion in breakout rooms
You will be prompted to return by the host
Large group debrief and feedback survey
# BREAKOUT GROUPS

## Slide 19
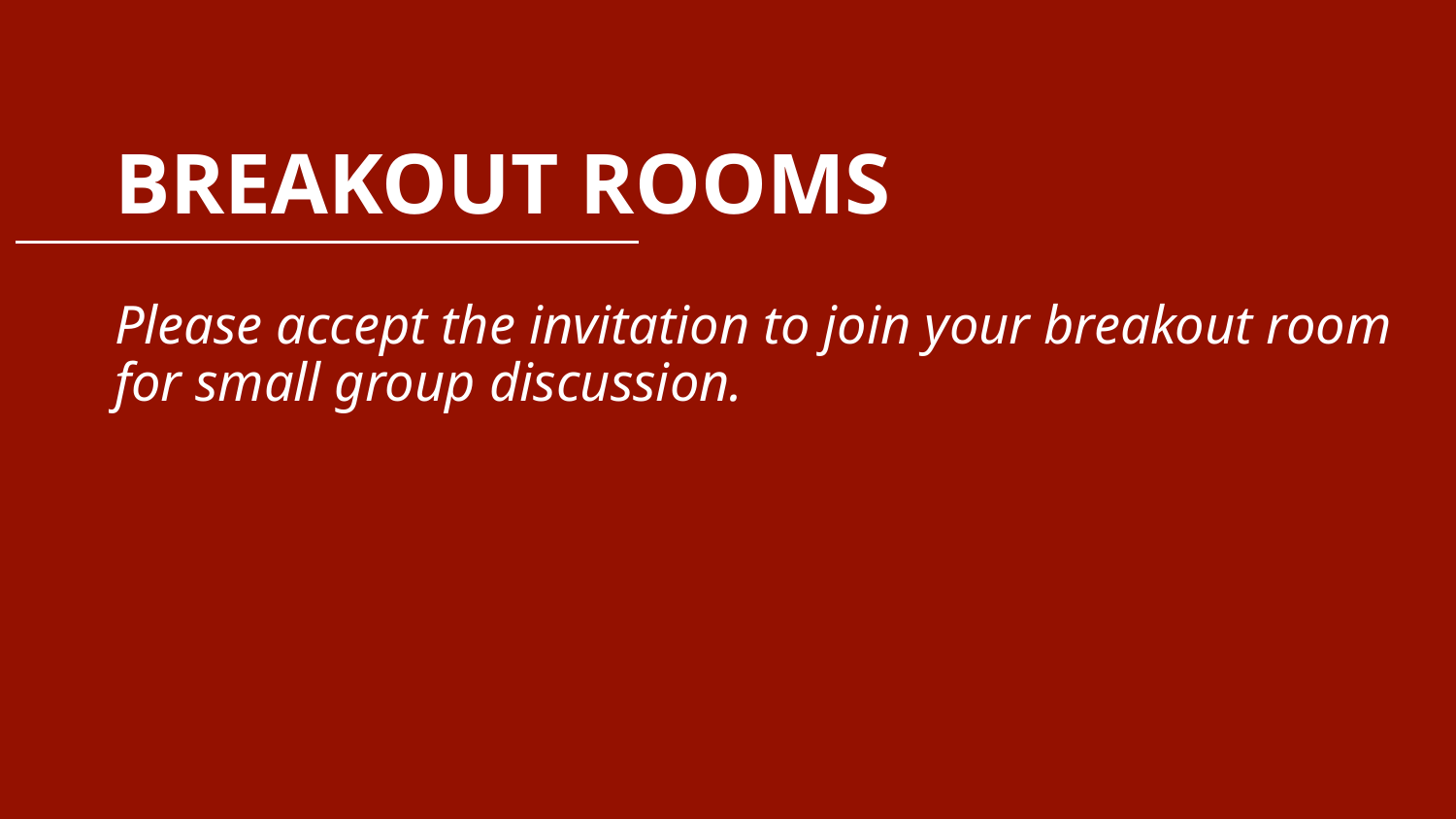

# BREAKOUT ROOMS
Please accept the invitation to join your breakout room for small group discussion.

## Slide 20
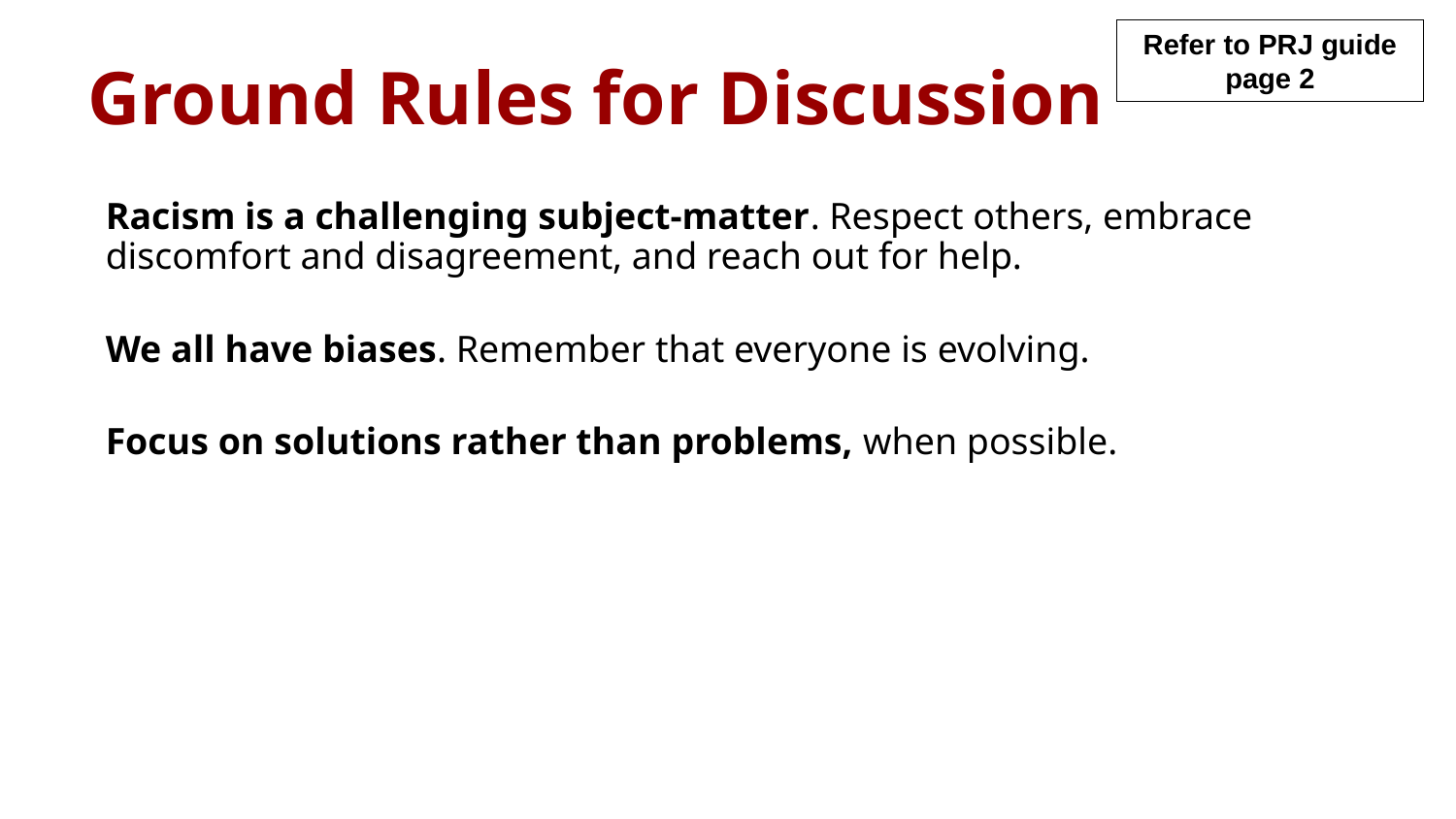

Refer to PRJ guide page 2
# Ground Rules for Discussion
Racism is a challenging subject-matter. Respect others, embrace discomfort and disagreement, and reach out for help.
We all have biases. Remember that everyone is evolving.
Focus on solutions rather than problems, when possible.

## Slide 21
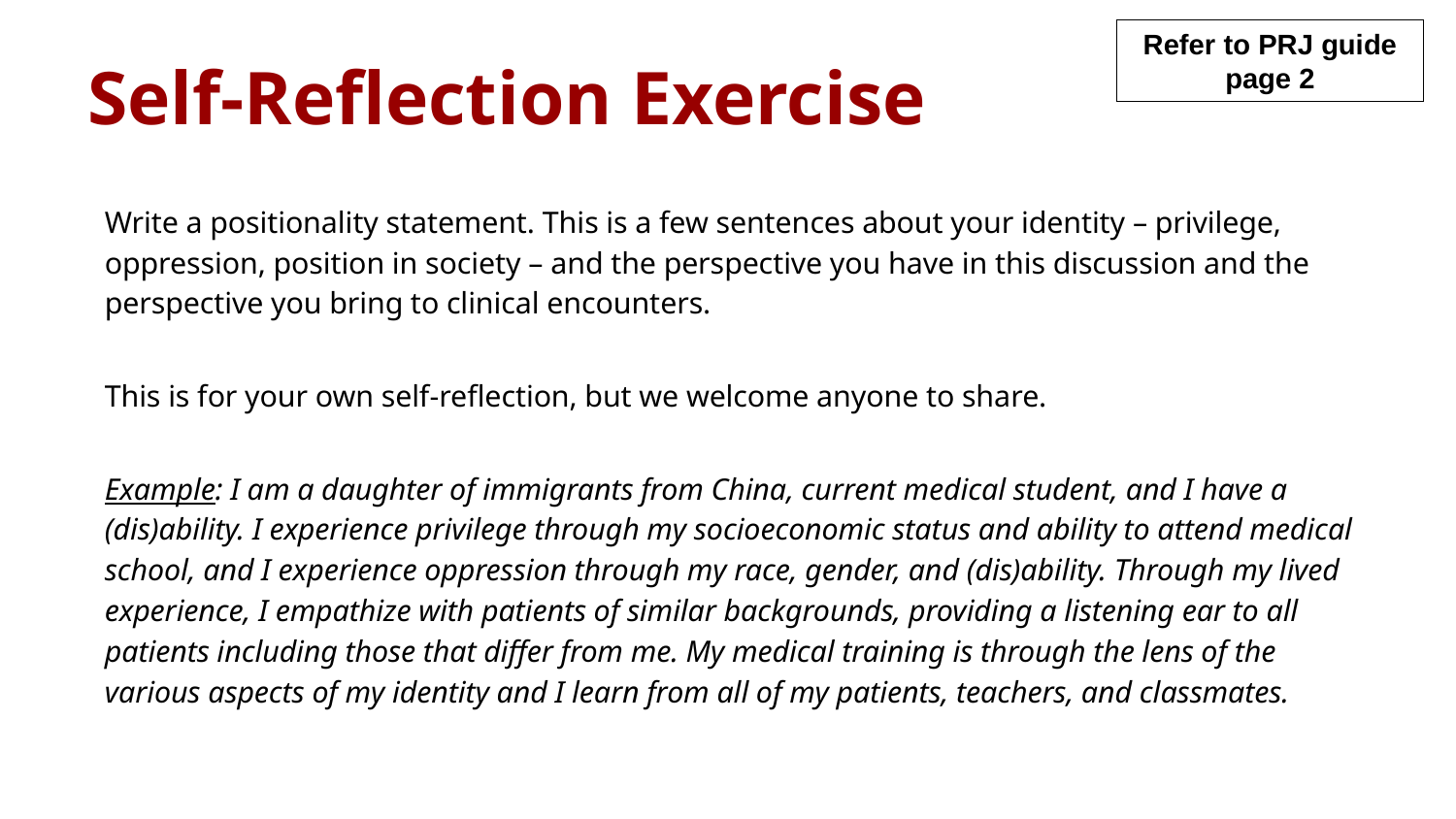

Refer to PRJ guide page 2
# Self-Reflection Exercise
Write a positionality statement. This is a few sentences about your identity – privilege, oppression, position in society – and the perspective you have in this discussion and the perspective you bring to clinical encounters.
This is for your own self-reflection, but we welcome anyone to share.
Example: I am a daughter of immigrants from China, current medical student, and I have a (dis)ability. I experience privilege through my socioeconomic status and ability to attend medical school, and I experience oppression through my race, gender, and (dis)ability. Through my lived experience, I empathize with patients of similar backgrounds, providing a listening ear to all patients including those that differ from me. My medical training is through the lens of the various aspects of my identity and I learn from all of my patients, teachers, and classmates.

## Slide 22
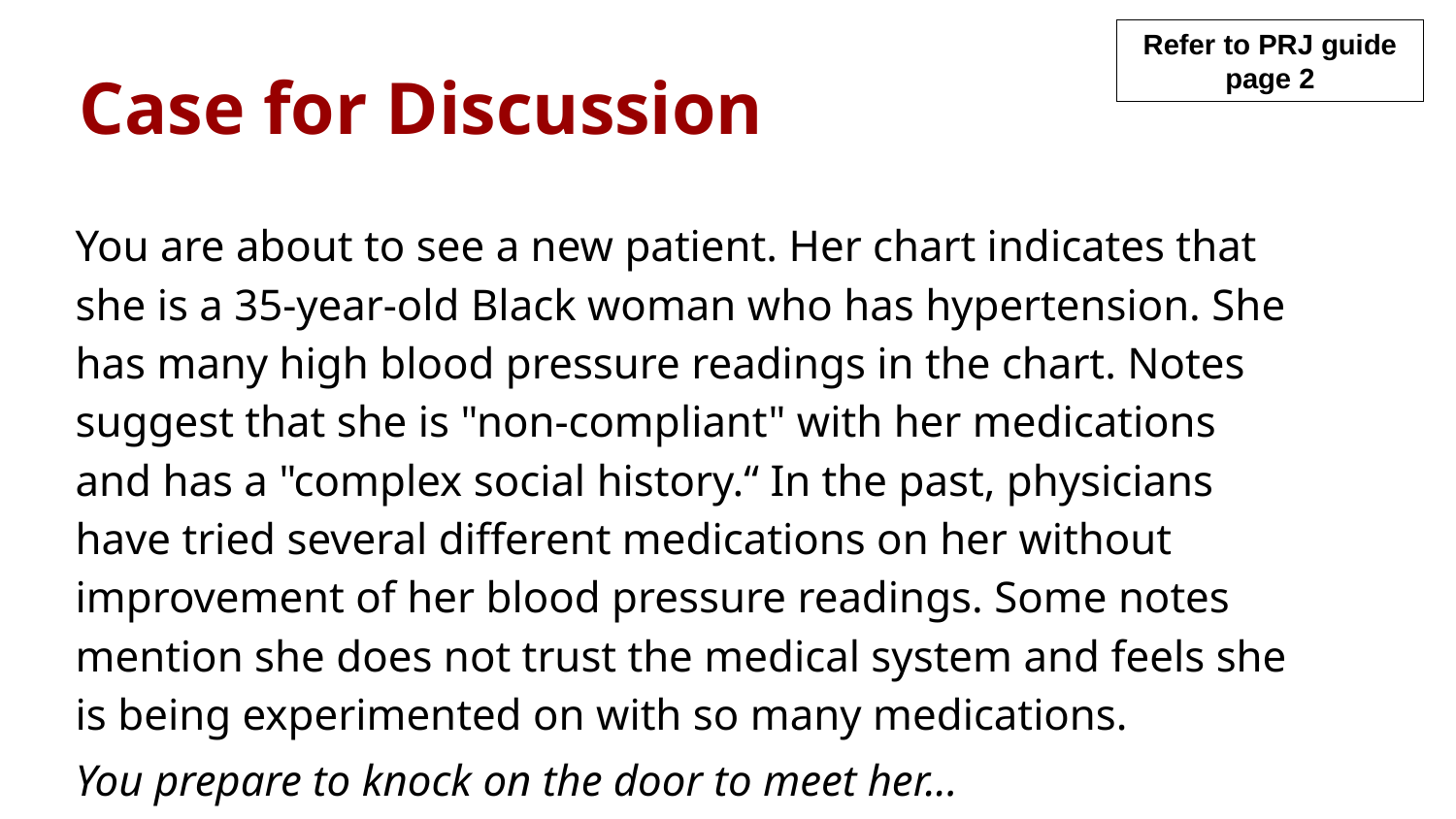

Refer to PRJ guide page 2
# Case for Discussion
You are about to see a new patient. Her chart indicates that she is a 35-year-old Black woman who has hypertension. She has many high blood pressure readings in the chart. Notes suggest that she is "non-compliant" with her medications and has a "complex social history.“ In the past, physicians have tried several different medications on her without improvement of her blood pressure readings. Some notes mention she does not trust the medical system and feels she is being experimented on with so many medications.
You prepare to knock on the door to meet her...

## Slide 23
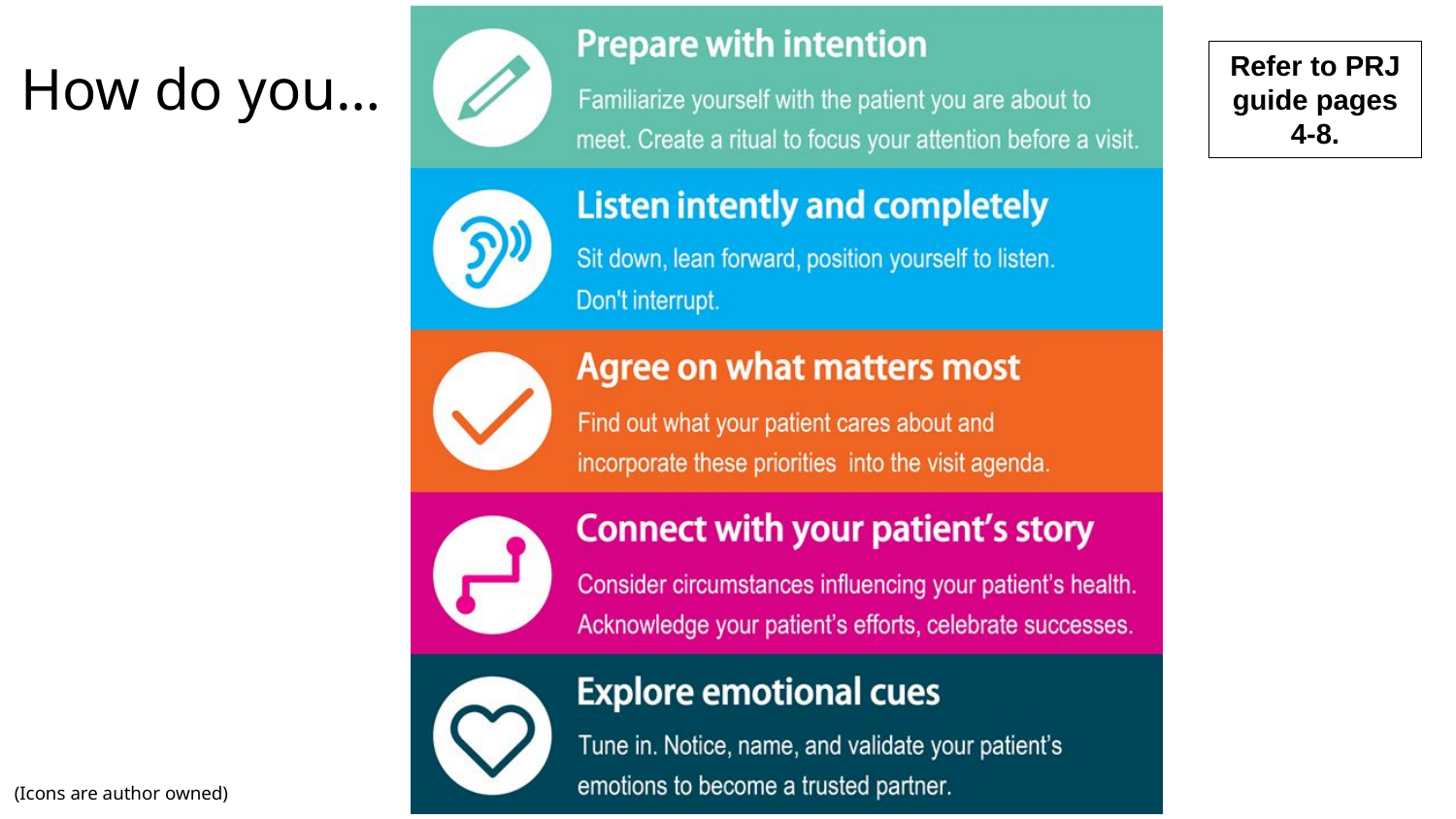

# How do you…
Refer to PRJ guide pages 4-8.
(Icons are author owned)

## Slide 24
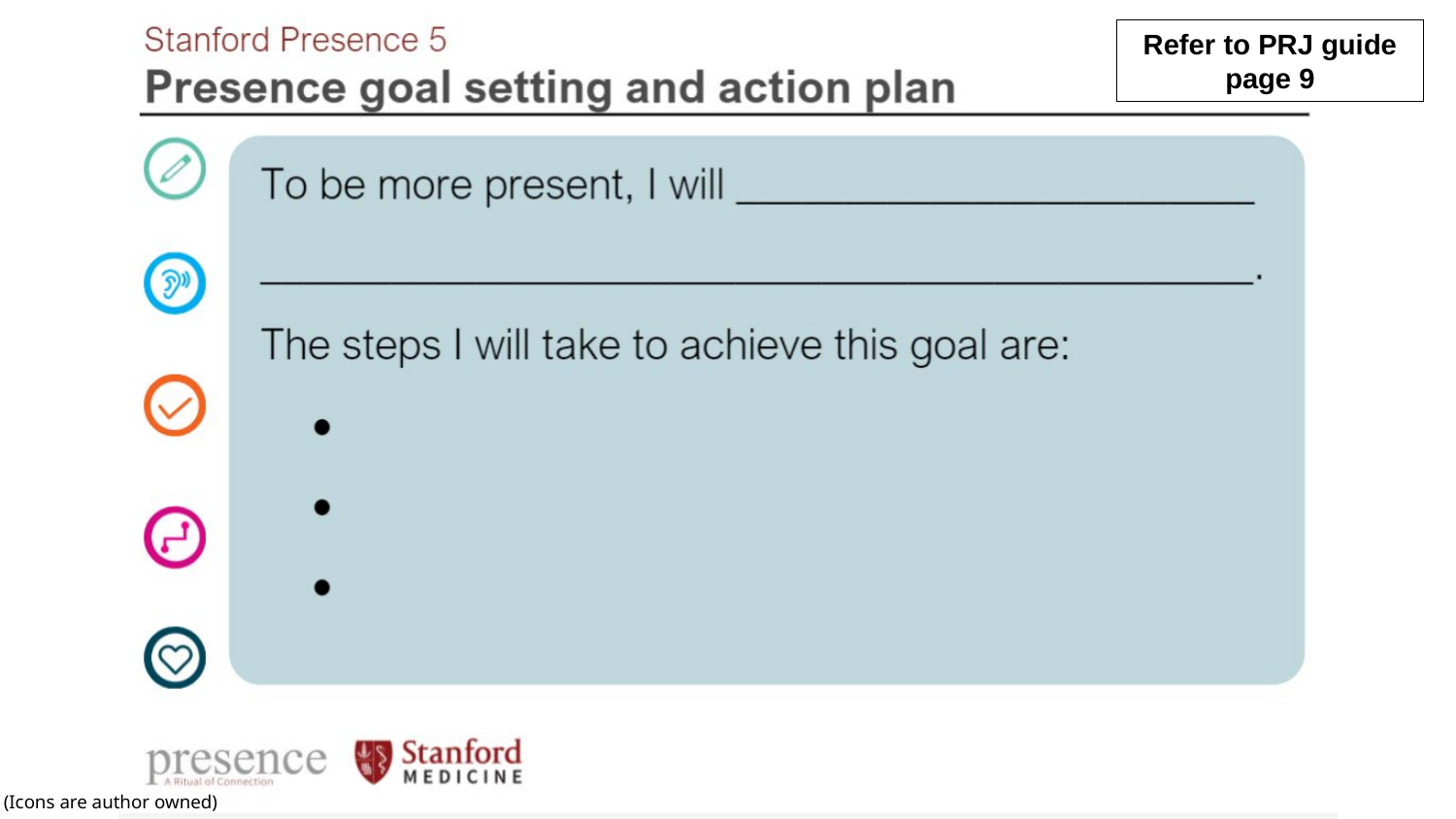

Refer to PRJ guide page 9
(Icons are author owned)

## Slide 25
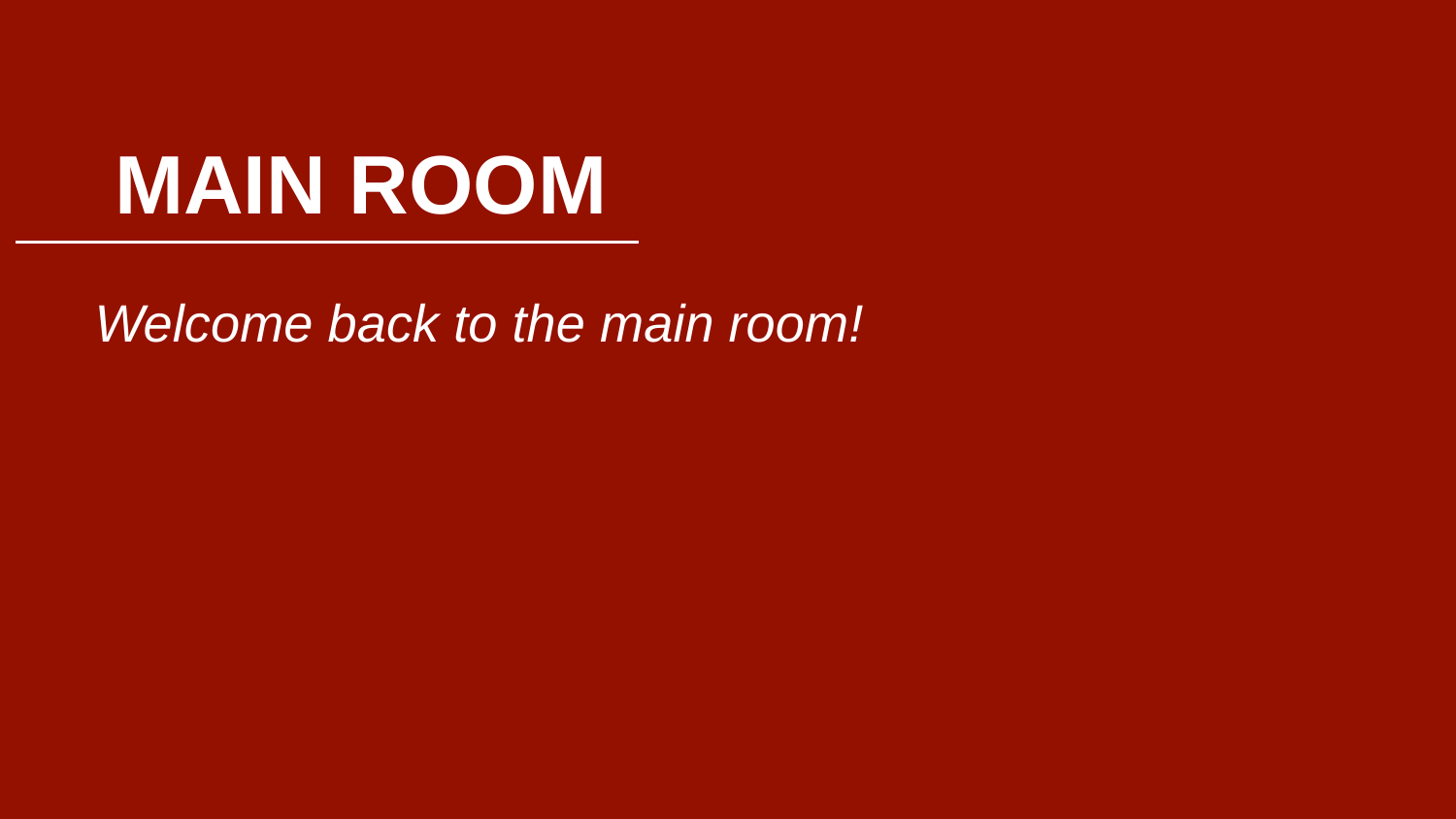

# MAIN ROOM
Welcome back to the main room!

## Slide 26
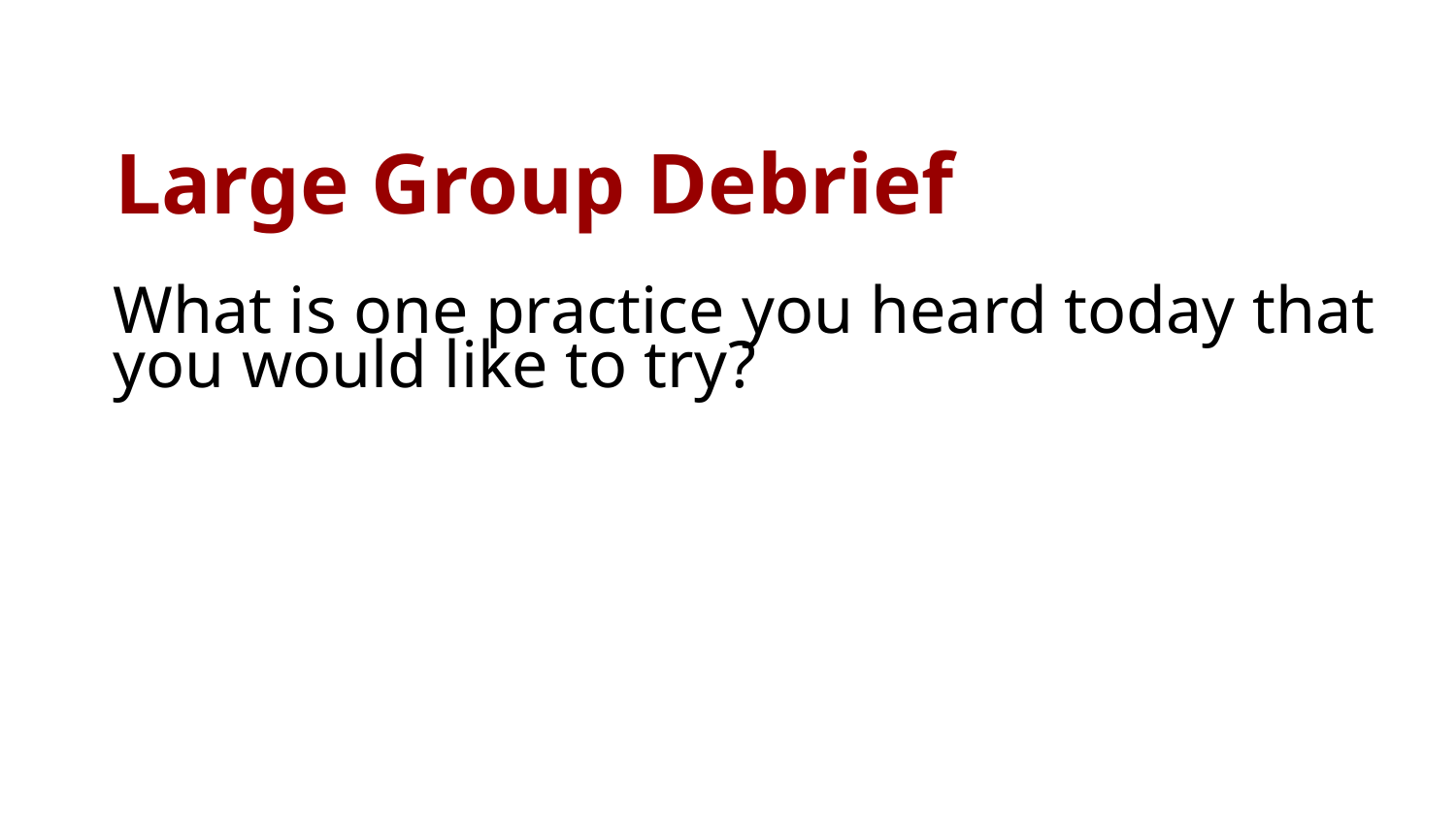

# Large Group Debrief
What is one practice you heard today that you would like to try?

## Slide 27
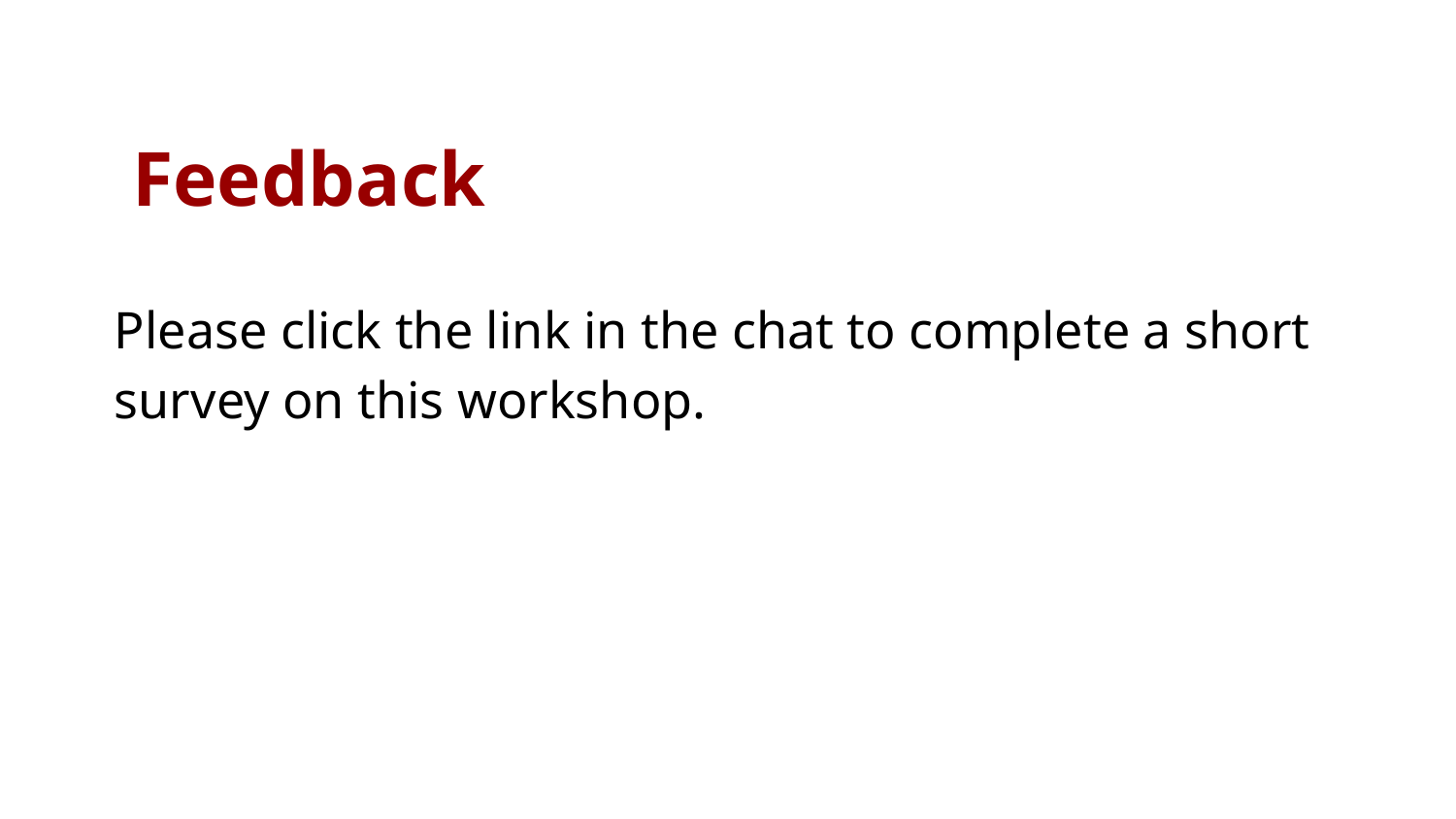

# Feedback
Please click the link in the chat to complete a short survey on this workshop.

## Slide 28
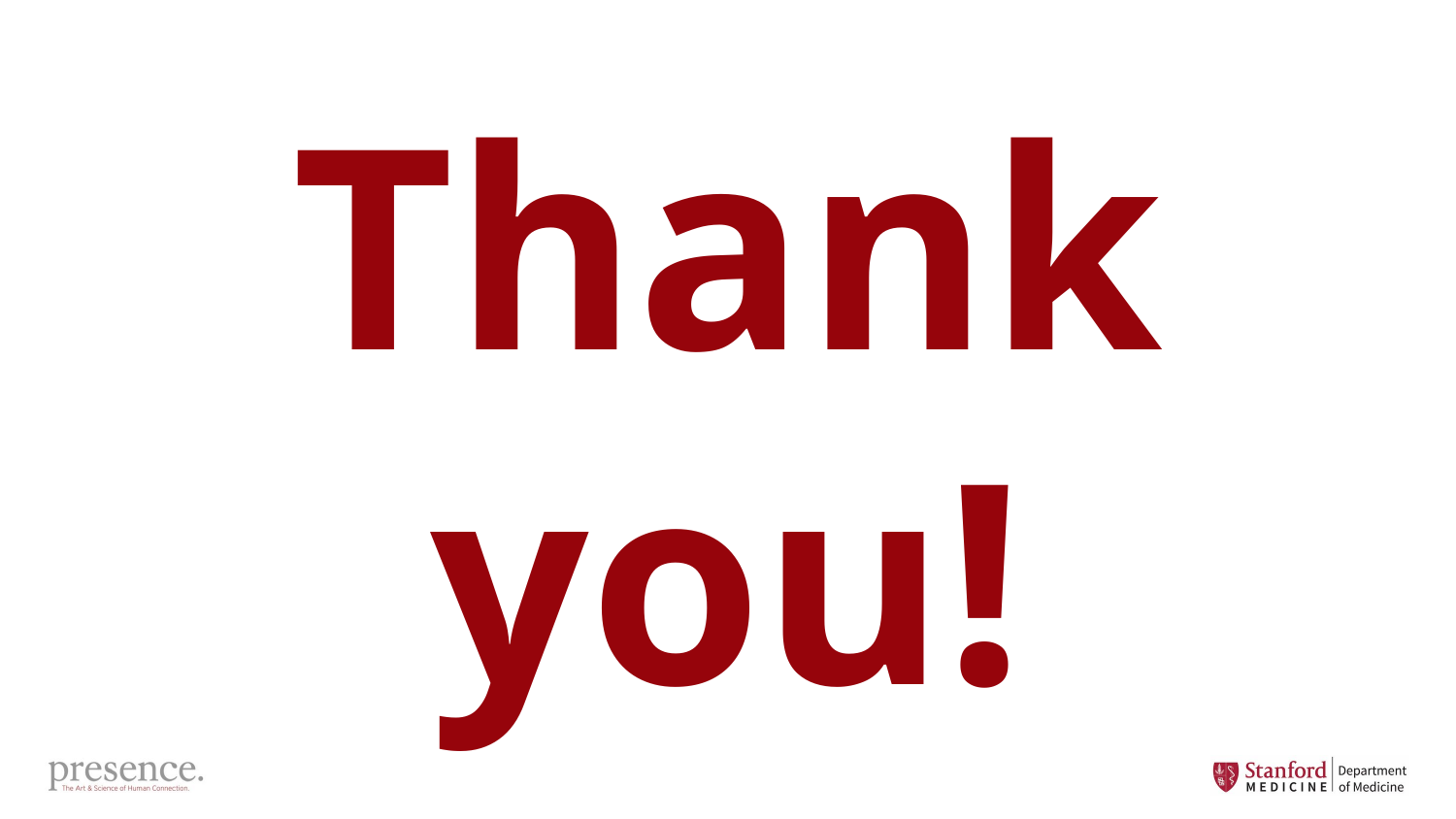

Thank you!
