# Supplementary material for: Presence 5 for Racial Justice Workshop: Fostering Dialogue Across Medical Education to Disrupt Anti-Black Racism in Clinical Encounters
Source: MedEdPORTAL. 2022 Feb 10;18:11227. doi: 10.15766/mep_2374-8265.11227 (PMC8828658; doi:10.15766/mep_2374-8265.11227)
Supplement: Supplementary file 1 — Presence 5 for Racial Justice Guide.docxIntroductory Didactic.pptxParticipant Resources.docxSurvey.docx [file mep_2374-8265.11227-s001.zip › D. Survey.docx]

**Presence 5 for Racial Justice Workshop Survey** (author owned)

**IDENTIFIERS**

Thank you for participating in the Presence 5 for Racial Justice Medical Education Feedback survey. These five “Presence 5” practices seek to foster meaningful connection between patient and clinicians. We are working on adapting this framework to promote racial justice within clinical education, with a specific focus on combating anti-Black racism. Please note that all information you provide will be de-identified and aggregated.

Name (first, last):

Email:

Institution:

DEMOGRAPHIC INFORMATION

Gender:

Woman Man

Non-Binary/Genderqueer

Write In:

Race/Ethnicity (select all that apply):

Black or African American (e.g. African American, Jamaican, Ethiopian, Haitian, etc.) American Indian or Alaska Native (e.g. Navajo Nation, Blackfeet Tribe, Mayan, Aztec, etc.) Asian (e.g. Chinese, Vietnamese, Indian, Lebanese, etc.)

Native Hawaiian or Pacific Islander (e.g. Kanaka Maoli, Samoan, Chamorro, etc.) Hispanic, Latinx, or Spanish Origin (e.g. Puerto Rican, Cuban, Salvadoran, etc.) White or Caucasian (e.g. German, Irish, Italian, etc.)

Write In:

What is your title/role at your institution?

What is your level of clinical practice?

Clinician/Faculty Medical Trainee

In which setting(s) is your clinical practice or training? (select all that apply)

Private Practice Academic Medical Center Inpatient

Outpatient

Community Health Center Veteran's Affairs (VA)

Write-in

Where is your clinical practice or training located?

Urban Setting

Rural Setting

Suburban Setting

If you are a trainee, what level of training are you currently at?

Medical school - 1st Year Resident - 3rd Year

Medical School - 2nd Year Resident - 4th+ Year

Medical School - 3rd Year Fellow - 1st Year

Medical School - 4th+ Year Fellow - 2nd Year

Resident - 1st Year/Intern Fellow - 3rd+ Year

Resident - 2nd Year Write-in (MPH, PhD, MBA, etc.)

How much time do you spend in direct patient care?

Clinical hours per week: 0 10 20 30 40 50 60 70 80 90 100

In what capacity do you teach or work with medical trainees? (select all that apply):

I precept medical trainees as an attending or senior resident/fellow I teach didactics for medical trainees

I do not teach or work with medical trainees

Write In

Please indicate what Diversity, Equity, and Inclusion efforts you have been a part of (select all that apply):

Leadership or Administration

Committee

Faculty hiring

Student recruitment

Research

Teaching/Curriculum Development Mentorship

Community engagement/programming Educational Workshops

Write In

WORKSHOP FEEDBACK

Below is the complete One-Pager on Presence for Racial Justice: 5 Practices. In the following section of this survey, you will be asked for your feedback about each practice one by one. Please read through each practice carefully and thoroughly, and provide feedback about how we can best improve the practice description or any of the example practices. Thank you!


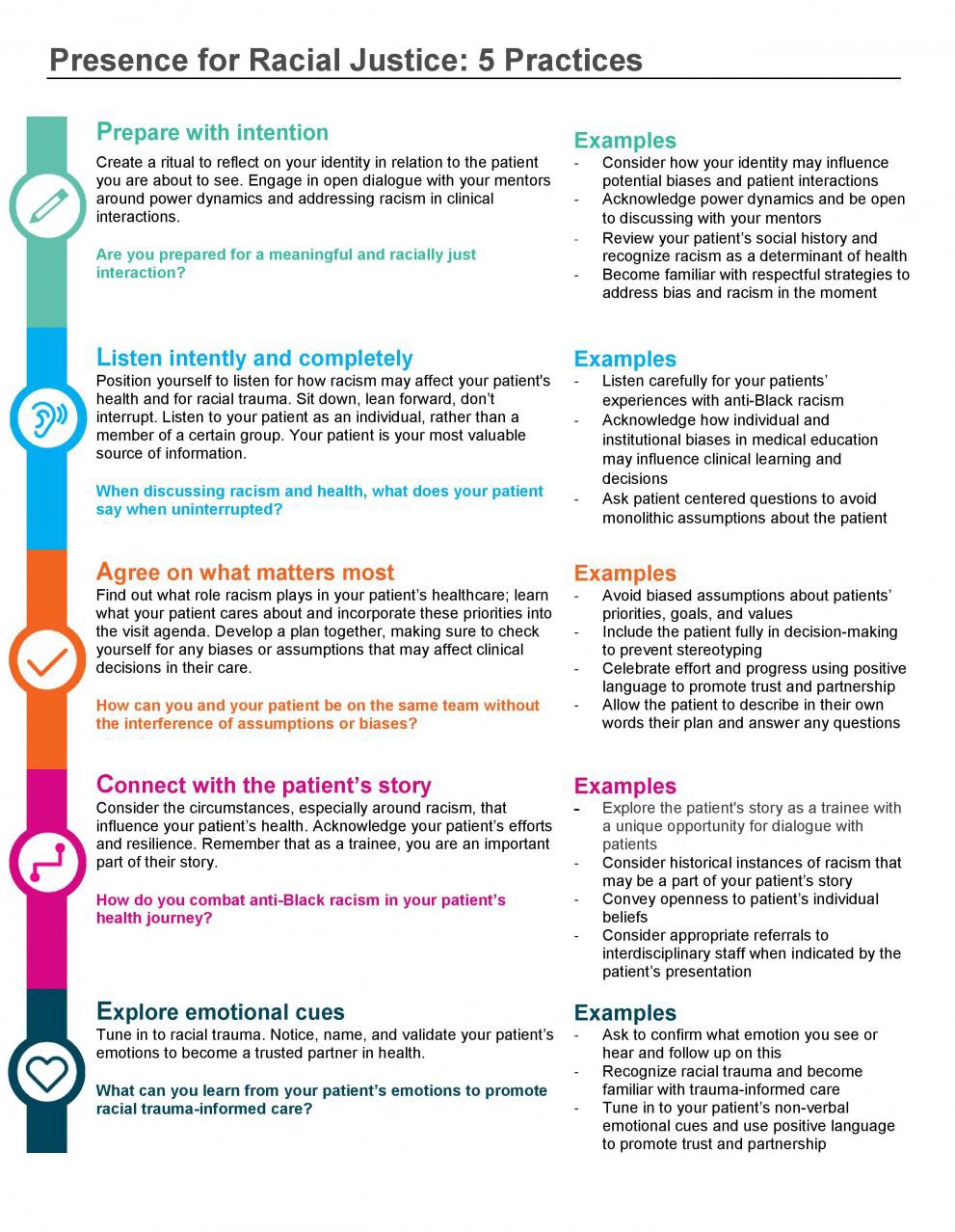


# Prepare with intention

Create a ritual to reflect on your identity in relation to the patient you are about to see. Engage in open dialogue with your mentors around power dynamics and addressing racism in clinical interactions.

**Are you prepared for a meaningful and racially just interaction?**

**Examples:**

Consider how your identity may influence potential biases and patient interactions Acknowledge power dynamics and be open to discussing with your mentors

Review your patient’s social history and recognize racism as a determinant of health Become familiar with respectful strategies to address bias and racism in the moment

What suggestions do you have to the practice description?

What suggestions do you have for the practice examples?

What specific phrases or examples of language do you use with patients for this practice?

# Listen intently and completely

Position yourself to listen for how racism may affect your patient's health and for racial trauma. Sit down, lean forward, don’t interrupt. Listen to your patient as an individual, rather than a member of a certain group. Your patient is your most valuable source of information.

**When discussing racism and health, what does your patient say when uninterrupted?**

**Examples:**

Listen carefully for your patients’ experiences with anti-Black racism

Acknowledge how individual and institutional biases in medical education may influence clinical learning and decisions

Ask patient centered questions to avoid monolithic assumptions about the patient

What suggestions do you have to the practice description?

What suggestions do you have for the practice examples?

What specific phrases or examples of language do you use with patients for this practice?

# Agree on what matters most

Find out what role racism plays in your patient’s healthcare; learn what your patient cares about and incorporate these priorities into the visit agenda. Develop a plan together, making sure to check yourself for any biases or assumptions that may affect clinical decisions in their care.

**How can you and your patient be on the same team without the interference of assumptions or biases?**

**Examples:**

Avoid biased assumptions about patients' priorities, goals, and values Include the patient fully in decision-making to prevent stereotyping

Celebrate effort and progress using positive language to promote trust and partnership Allow the patient to describe in their own words their plan and answer any questions

What suggestions do you have to the practice description?

What suggestions do you have for the practice examples?

What specific phrases or examples of language do you use with patients for this practice?

# Connect with the patient’s story

Consider the circumstances, especially around racism, that influence your patient’s health. Acknowledge your patient’s efforts and resilience. Remember that as a trainee, you are an important part of their story.

**How do you combat anti-Black racism in your patient’s health journey?**

**Examples:**

Explore the patient's story as a trainee with a unique opportunity for dialogue with patients

Consider historical instances of racism that may be part of your patient's story Convey openness to patient's individual beliefs

Consider appropriate referrals to interdisciplinary staff when indicated by the patient's presentation

What suggestions do you have to the practice description?

What suggestions do you have for the practice examples?

What specific phrases or examples of language do you use with patients for this practice?

# Explore emotional cues

Tune in to racial trauma. Notice, name, and validate your patient’s emotions to become a trusted partner in health.

**What can you learn from your patient’s emotions to promote racial trauma-informed care?**

**Examples:**

Ask to confirm what emotions you see or hear and follow up on this Recognize racial trauma and become familiar with trauma-informed care

Tune in to your patient's non-verbal emotional cues and use positive language to promote trust and partnership

What suggestions do you have to the practice description?

What suggestions do you have for the practice examples?

What specific phrases or examples of language do you use with patients for this practice?

**Workshop Feedback**

What were strengths of the workshop and discussion group structure? How could the workshop and discussion group structure be improved?

What did you find most valuable about the content and evidence presented during discussion? How could the discussion content and evidence be improved?
